# Estimating the impact of nutritional transition and ending hunger on tuberculosis in 12 high-burden countries: a model-based scenario analysis

**DOI:** 10.1136/bmjgh-2024-018839

**Published:** 2025-12-25

**Authors:** Chieh-Yin Wu, Chu-Chang Ku, Christopher Finn McQuaid, Knut Lönnroth, J Peter Cegielski, James Bentham, Majid Ezzati, Hsien-Ho Lin

**Affiliations:** 1 National Taiwan University, Taipei City, Taiwan; 2 Department of Infectious Disease Epidemiology and Dynamics, London School of Hygiene & Tropical Medicine, London, UK; 3 Department of Global Public Health, Karolinska Institute, Stockholm, Sweden; 4 Department of Epidemiology, Emory University Rollins School of Public Health, Atlanta, Georgia, USA; 5 School of Mathematics, Statistics and Physics, Newcastle University, Newcastle upon Tyne, UK; 6 School of Public Health, Imperial College London, London, UK; 7 MRC-PHE Centre for Environment and Health, London, UK

**Keywords:** Tuberculosis, Nutrition

## Abstract

**Background:**

Nutrition is a critical determinant of tuberculosis (TB), providing a protective effect at high body mass index (BMI) and incurring an increased risk of TB disease at low BMI. Global nutritional transition and interventions to end hunger could directly affect the TB epidemic in high TB burden countries.

**Methods:**

We constructed dynamic TB transmission models for 12 high TB burden countries with low HIV prevalence. We explicitly accounted for the effects of BMI on TB disease progression and treatment outcomes using a meta-analysis of longitudinal cohort studies, incorporating the effect of BMI mediated through diabetes. The models were calibrated to historical trends in TB epidemiology and mean BMI. We estimated potential changes in TB incidence and mortality between 2015 and 2030 under different scenarios of population nutrition.

**Findings:**

Compared with a scenario where mean BMI remained at 2015 levels, if past trends in mean BMI continued then by 2030 TB incidence and mortality would decline by a cumulative 14.7% (95% credible interval: 12.7%–16.7%) and 15.6% (12.5%–19.2%), respectively. In comparison, achieving zero hunger by 2030 would reduce incidence and mortality by 32.0% (20.0%–43.8%) and 37.3% (26.1%–49.6%), respectively. If past trends continued and zero hunger was also achieved, incidence and mortality would be reduced by 38.2% (27.0%–49.1%) and 42.4% (32.1%–53.5%), respectively, equivalent to preventing 20.6 million people developing TB disease and averting 5.4 million TB deaths over 15 years in the 12 high-burden countries.

**Conclusions:**

Nutrition transitions and interventions to end hunger could have a major impact on the future epidemiology of TB in high-burden countries. Investment is urgently required to implement and scale up nutritional interventions.

**WHAT IS ALREADY KNOWN ON THIS TOPIC**

- Undernutrition increases the risk of tuberculosis (TB) progression and death, while higher body mass index (BMI) provides a protective effect. A global nutritional transition is underway, with rising obesity in some regions and persistent undernutrition in many high TB burden countries.

**WHAT THIS STUDY ADDS**

- This study provides the first multicountry, model-based assessment of how nutrition transitions, including rising BMI and declining undernutrition, together with the mediating effect of diabetes, may affect future TB incidence and mortality. Our findings indicate that achieving zero hunger alongside continued BMI increases could avert over 20 million TB cases and 5 million deaths by 2030 across 12 high TB burden countries.

**HOW THIS STUDY MIGHT AFFECT RESEARCH, PRACTICE OR POLICY**

- The findings underscore the importance of integrating nutritional strategies into TB control efforts. Addressing undernutrition and supporting nutrition goals could significantly reduce TB burden and accelerate progress towards the End TB Strategy. National TB programmes should align with broader health and development initiatives, including the Sustainable Development Goals.

## Introduction

 Tuberculosis (TB) continues to be the most lethal infectious disease globally. The End TB Strategy set by the WHO aims to end the global TB epidemic by achieving a 90% reduction in TB mortality and 80% reduction in TB incidence by 2030,^1^ where the role of TB determinants such as nutrition is critical.^2^ Nutritional status has long been recognised as a major determinant of TB burden; being underweight increases risk of TB disease over threefold,^3 4^ while being overweight or obese reduces risk.^5 6^ In general, an inverse log linear relationship has been demonstrated between body mass index (BMI) and TB incidence.^7 8^ In contrast, diabetes, which is also associated with a twofold increased risk of TB disease,^9^ is strongly associated with a high BMI and obesity.^10^ Between them, at least one in four TB cases globally is thought to be attributable to either undernutrition or diabetes.^11^ Both undernutrition and diabetes are separately associated with an increased risk of poor TB treatment outcomes and death due to TB.^12 13^

If current trends in global BMI continue, the world faces an epidemic of severe obesity; nevertheless, undernutrition will also remain widely prevalent in high TB burden countries, particularly in South Asia and parts of Africa.^14^ This transition in global nutritional status, aligning with two key risk factors (undernutrition and diabetes) for TB, has the potential to substantially impact future epidemiology of TB in high burden countries. In particular, the United Nations Sustainable Development Goal 2 of ‘Zero Hunger’ has direct and potentially significant consequences for ending the TB epidemic.^15^ In a recent cluster-randomised controlled trial (RATIONS) in India, nutritional intervention was associated with a substantial (39%–48%) reduction of TB incidence among household contacts of TB patients.^16^ Subsequently, a mathematical model incorporating the results from the RATIONS trial revealed that nutritional supplementation could reduce TB incidence in South-East Asian countries, and the benefits would be much larger if the intervention was expanded from household contacts to the general population.^17^ Previous research has primarily focused on addressing undernutrition, with limited attention to the full spectrum of nutritional transition, which includes both overnutrition and undernutrition. Our model-based analysis aims to fill this gap by quantifying the impact of various population nutrition scenarios on progress towards the WHO’s End TB Strategy targets. We used dynamic TB transmission models with explicit BMI strata to assess the impact of global nutritional transition and nutritional interventions on TB epidemiology in selected high TB burden countries between 2015 and 2030.

## Methods

### Analytical settings and model

Among the 30 high TB burden countries listed by WHO^18^ in 2015, 20 have the highest absolute number of TB cases, from which we modelled 12, excluding those with high prevalence of HIV infection (>1%) and North Korea with high uncertainty of TB and BMI estimates. These countries accounted for 64.2% of total incident TB cases globally and included Bangladesh, Brazil, Cambodia, China, India, Indonesia, Myanmar, Pakistan, Philippines, Russia, Thailand and Viet Nam.

**Figure 1:**
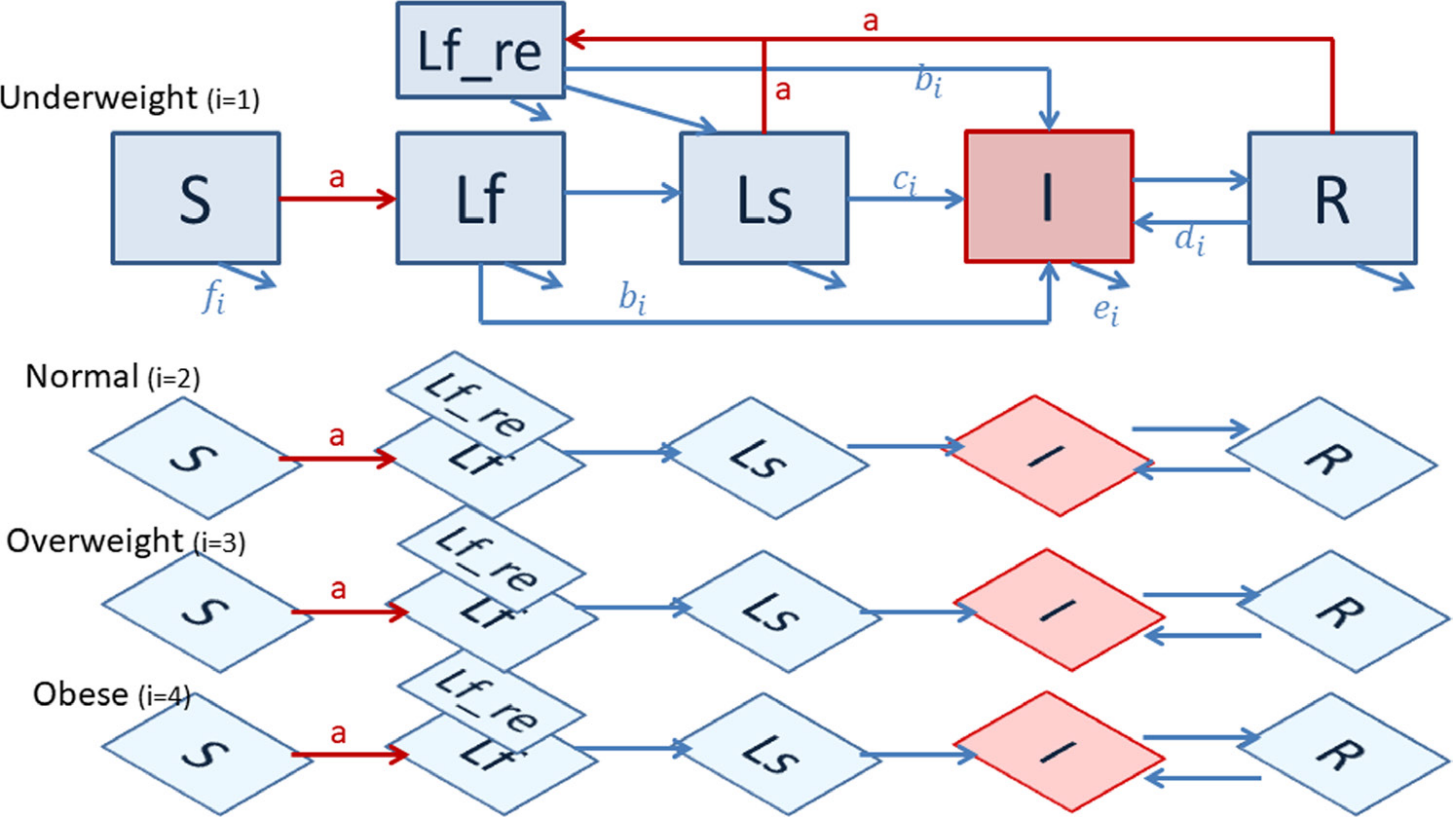
Dynamic compartmental tuberculosis transmission model structure, stratified by body mass index (BMI). *S: Susceptible, Lf: Fast latent Infection, LF_re: Fast latent reinfection, Ls: Slow latent infection, I: Active disease, R: Recovered. **a=infection (red lines: force of infection, which is determined by prevalence of infectious individuals (**I**) from all four BMI levels combined, b=BMI-specific primary progression from fast infection to disease, c=BMI-specific reactivation from slow infection to disease, d=BMI-specific relapse from recovered to disease, e=BMI-specific mortality of TB patients, f=BMI-specific general mortality from all compartments.

For each country, we constructed a dynamic compartmental model of TB transmission in the adult (>15 years old) population, incorporating TB natural history (figure 1 and online supplemental appendix 1), case detection rate and treatment success rate at the country level (online supplemental appendix 2). We further divided the population into four levels by BMI, including underweight (BMI <18.5), normal weight (18.5≤BMI<25), overweight (25≤BMI<30) and obese (BMI ≥30). For each BMI category, we considered six TB states: susceptible, fast infection, slow infection, pulmonary active disease, recovered and fast infection after reinfection. Each BMI submodel ran separately but was connected by a common force of infection (driven by all prevalent TB cases in the population) assuming homogeneous mixing. Population-level inference from the TB model was implemented by weighted average of outputs from the four BMI submodels using the estimated prevalence of BMI categories (see below).

We projected and compared changes in TB epidemiology under different BMI scenarios in 2015–2030. The baseline year of projection was set to 2015, corresponding to the starting year of the End TB Strategy. We decided to ignore the impact of COVID-19 on TB and BMI in this analysis. Our analytic scheme and time horizon was justified since the main purpose of the study was to understand and quantify the potential and counterfactual impact of nutritional transition on TB epidemiology in the context of the End TB Strategy.

### Incorporating BMI and diabetes

We incorporated the effects of BMI on different stages of TB disease and mortality in light of previous studies and systematic reviews (figure 1 and online supplemental table). First, there is limited evidence on the association between BMI and the risk of TB infection. A systematic review of cross-sectional studies revealed no significant association between nutritional status and the results from tuberculin skin test or interferon gamma release assay.^19^ Second, there is evidence of a strong and inverse association between BMI and risk of active TB from large population-based cohort studies.^5^,^720 21^ Given the lack of association between BMI and TB infection, we assumed that the inverse association between BMI and active TB was due to the impact of BMI on the risk of progression from TB infection to active disease. Third, there is evidence that the case fatality rate of TB patients is higher in the underweight.^12 22 23^ Lastly, there is evidence of a U-shape dose-response association between BMI and general mortality.^24^

We considered the total effect of BMI on TB, including the direct effect not mediated through diabetes and the indirect effect mediated through diabetes (online supplemental figure). This was achieved by using relative risk estimates between BMI and TB from longitudinal cohort studies that did not adjust for diabetes in the multivariable analysis (online supplemental table).^5 6^

Estimates of country-specific mean BMI in 1980–2015 were derived from a systematic review and data pooling of 1698 population-based measurement studies using a Bayesian hierarchical model.^25^ Using the cross-walk model from the same study, we extrapolated the mean BMI to the prevalence of categorical BMI in different age and sex groups (online supplemental appendix 3). All analyses of BMI were stratified by age and sex. Country-specific BMI prevalence was weighted by age and sex population distribution before input to the TB model.

### Model calibration

Country-specific models were calibrated to WHO estimates of TB incidence from 2006 to 2015^26^ using an Approximated Bayesian Computation rejection algorithm and the prior distributions in table 1^27^ In brief, a distance metric was measured using the Euclidean distance of simulated incidence from 2006 to 2015 from WHO country-specific estimates. The 5% of the distance from 100 sample runs was set to be the tolerance. If the distance from one simulation is less than or equal to the tolerance, the current parameter values were stored and the counter was updated. The loop continued until 10 000 accepted posterior parameter sets were collected (details in online supplemental appendix 1.2).

**Table 1:** Scenarios of BMI trend

Scenario	Description	References
Current level	Mean BMI remains the same as 2015. As a result, the proportion of underweight, normal, overweight and obese population remains unchanged after 2015.	WHO non-communicable disease target 7 for obesity^32^
Continuing trend	BMI trends between 2000 and 2015 continue, but not above BMI=38. The proportion of underweight, normal, overweight and obese population is estimated using the cross walk formula from the systematic analysis of BMI.	Systematic analysis of BMI by the NCD-RisC^25^
Current level and zero hunger	The prevalence of overweight and obesity remains unchanged after 2015, and the prevalence of underweight decreases linearly to zero from 2015 to 2030, where all underweight are assumed to become normal weight.	WHO non-communicable disease target 7 for obesity Sustainable Development Goal 2 Zero Hunger
Continuing trend and zero hunger	BMI trends between 2000 and 2015 continue, but not above BMI=38. Prevalence of underweight decreases linearly to zero from 2015 to 2030, where all underweight individuals are assumed to become normal weight.	Systematic analysis of BMI by the NCD-RisC Sustainable Development Goal 2 Zero Hunger

BMI, body mass index.

### Future scenarios

For the period after 2015, four scenarios of future BMI trajectory were investigated (table 1 and figure 1): Scenario 1 (‘current level’) the base case scenario where mean BMI remains unchanged in each country after 2015; Scenario 2 (‘continuing trend’) existing trends in mean BMI between 2000 and 2015 continue into the future; Scenario 3 (‘current level and zero hunger’) the proportion of the population overweight or obese remains the same, but the proportion of underweight decreases linearly to zero by 2030, where all underweight are assumed to become normal weight; Scenario 4 (‘continuing trend and zero hunger’) the proportion of overweight and obesity increases following the existing trends in mean BMI, then the proportion of the population underweight is set to decrease linearly to zero by 2030, where all underweight are assumed to become normal weight. We assumed no introduction of new technologies or interventions for TB, and that current TB intervention coverage and quality remained constant after 2015, since the focus of the analysis was on the impact of nutritional changes and TB. We estimated changes in TB incidence and mortality under different BMI scenarios from 2015 to 2030.

### Uncertainty and sensitivity analysis

The Bayesian calibration approach in our analysis accounted for uncertainty of all input parameters. Of the 100 000 posterior simulations from the Bayesian analysis, we reported the mean as the central estimates and their 2.5th and 97.5th percentiles as the 95% credible intervals (CrI). We also conducted one-way sensitivity analyses by changing each parameter to see how it affected the main results. This was done by repeating the simulations and changing one input parameter at a time to its highest or lowest value (posterior mean±one IQR) while holding other parameter values at their posterior set closest to the mean value.

## Results

**Figure 2:**
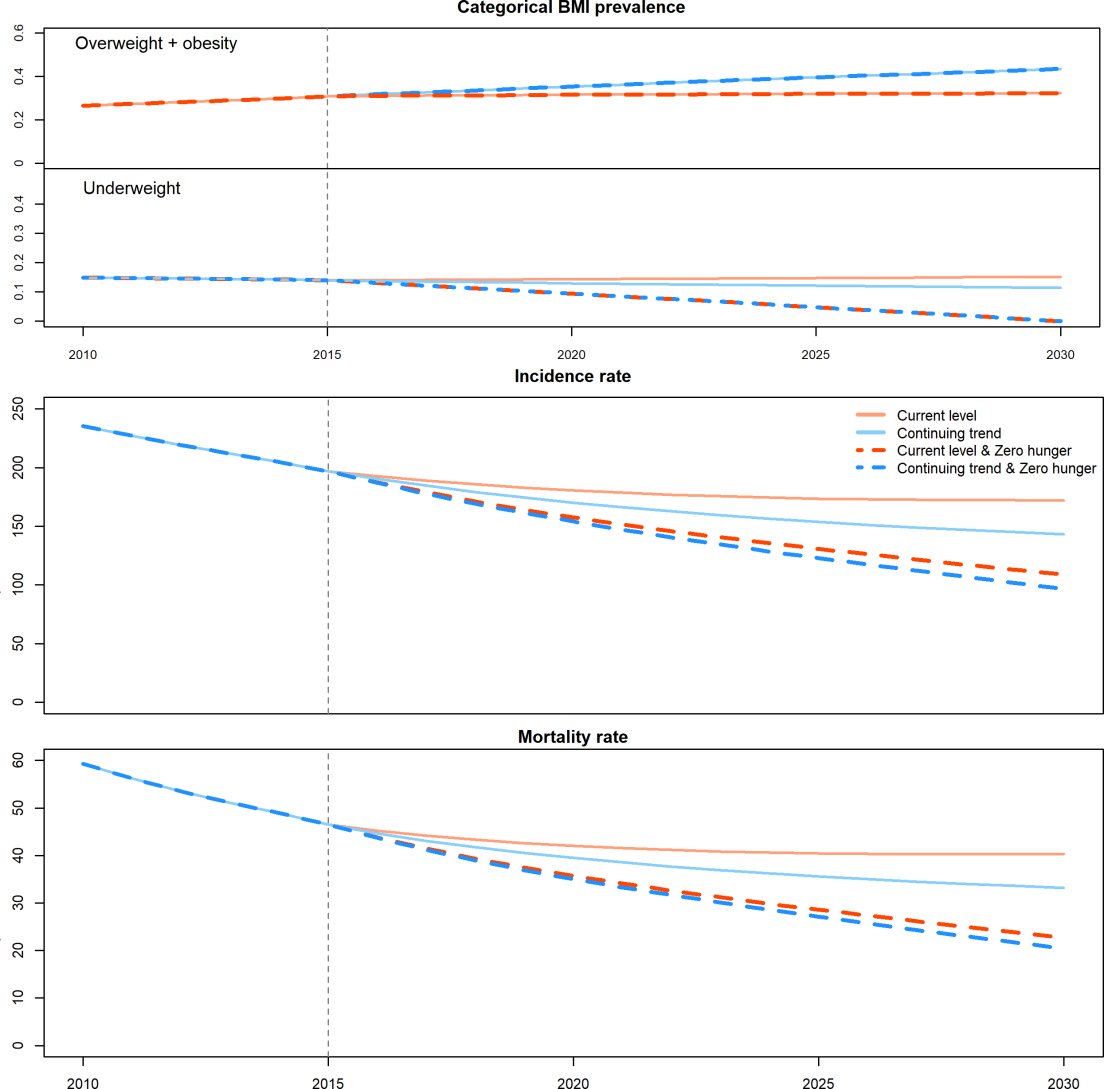
Categorical BMI prevalence (upper panel), projected incidence rate (middle panel) and mortality rate (lower panel) for the population-weighted mean of 12 high tuberculosis burden countries in different future scenarios. BMI, body mass index.

In the base case (current level) scenario, the population-weighted mean TB incidence and mortality of the 12 countries declined slowly and gradually levelled off (figure 2). Between 2015 and 2030, mean TB incidence decreased from 196.9 (credible interval, CrI: 165.8–232.8) to 172.1 (CrI: 133.6–223.2) per 100 000 and TB mortality decreased from 46.5 (CrI: 28.5–72.7) to 40.3 (CrI: 23.6–67.7) per 100 000. This was equivalent to a fall in incidence and mortality of 12.9% (CrI: 2.76%–23.6%) and 14.0% (CrI: 3.7%–23.2%), respectively.

**Figure 3:**
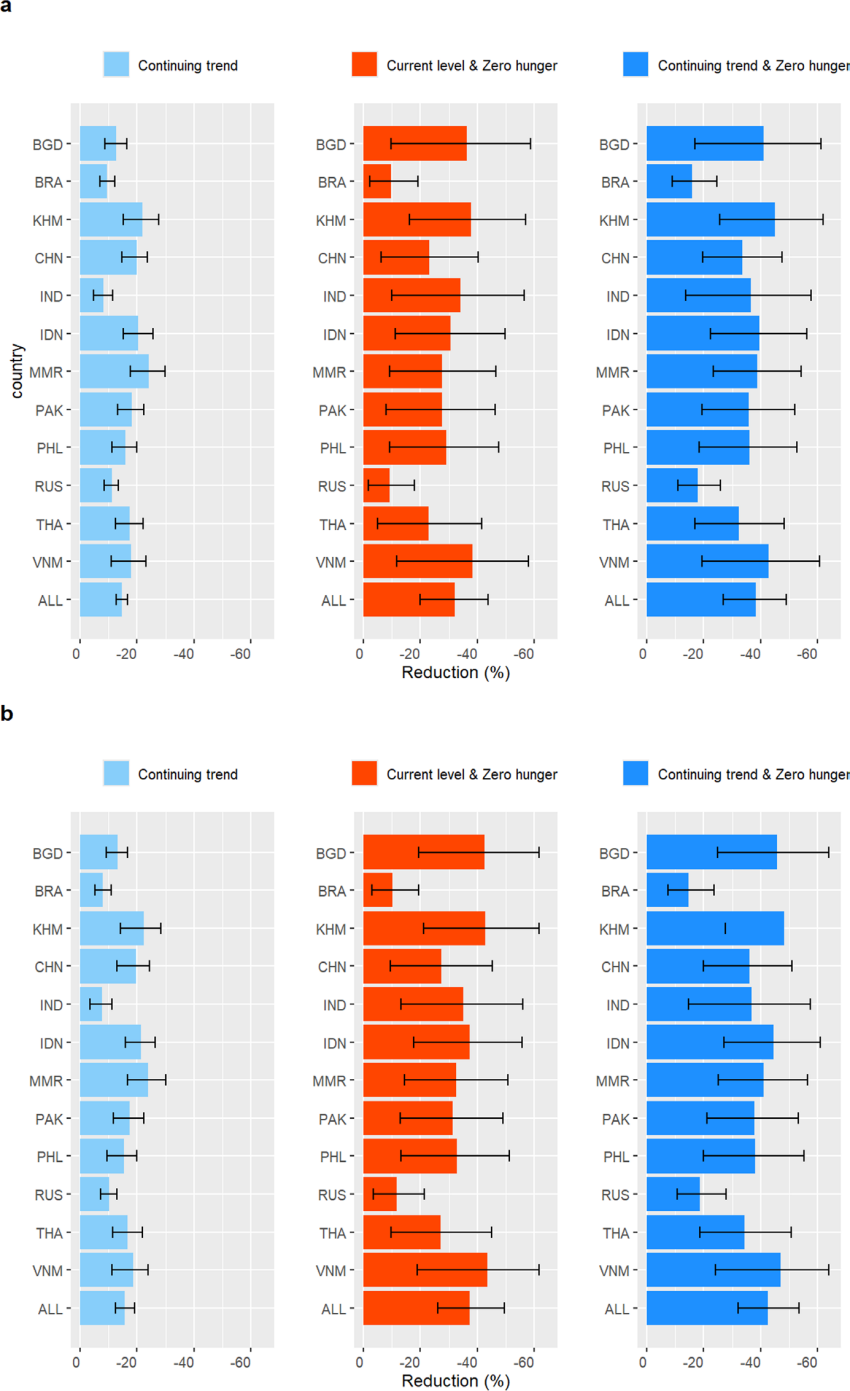
Cumulative reduction in tuberculosis (**a**) incidence and (**b**) mortality (from 2015 to 2030) under different scenarios of nutritional transition compared with the current level scenario in 12 high TB burden countries. BGD, Bangladesh; BRA, Brazil; CHN, China; IDN, Indonesia; IND, India; KHM, Cambodia; MMR, Myanmar; PAK, Pakistan; PHL, Philippines; RUS, Russian Federation; TB, tuberculosis; THA, Thailand; VNM, Viet Nam.

In the continuing trend scenario, the prevalence of overweight and obesity kept increasing and the prevalence of underweight decreased linearly (figure 2, upper panel). In this scenario, weighted TB incidence and mortality in 2030 compared with 2015 decreased by an additional 14.7% (CrI: 12.7%–16.7%) and 15.6% (CrI: 12.5%–19.2%) compared with the current level scenario (figure 2, middle and lower panel). Myanmar (incidence: 24.0%, 95% CrI 17.8%–29.9%; mortality: 23.9%, 95% CrI 16.7%–30.1%), Cambodia (21.9%, 15.2%–27.5%; 22.3%, 14.1%–28.3%) and Indonesia (20.5%, 15.1%–25.6%; 21.4%, 15.9%–26.5%) were the countries with the largest projected decline in incidence and mortality (figure 3).

In the current level and zero hunger scenario, the weighted TB incidence and mortality in 2030 compared with 2015 decreased by an additional 32.0% (CrI: 20.0%–43.8%) and 37.3% (CrI: 26.1%–49.6%) compared with the current level scenario. The largest decline of incidence occurred in Vietnam (38.2%, 11.7%–58.1%), Cambodia (37.9%, 16.2%–57.0%) and Bangladesh (36.4%, 9.6%–58.6%) (figure 3). In the continuing trend and zero hunger scenario, the additional decline of TB incidence and mortality by 2030 was the optimal, reaching 38.2% (CrI: 27.0%–49.1%) and 42.4% (CrI: 32.1%–53.5%), respectively. This is equivalent to preventing cumulatively 20.6 million TB cases and averting 5.4 million TB deaths in the 12 countries between 2015 and 2030 (table 2). The largest additional reductions in TB incidence and mortality were seen in Cambodia (45.0%, 25.7%–61.9%; 48.4%, 27.7%–65.6%), Vietnam (42.9%, 19.4%–60.6%; 47.0%, 24.0%–63.8%) and Bangladesh (41.0%, 17.0%–61.1%; 45.9%, 25.0%–63.9%); the smallest decline was observed in Brazil (15.9%, 8.9%–24.7%; 14.7%, 7.6%–23.6%) and Russia (17.8%, 10.9%–25.8%; 18.7%, 10.7%–27.8%) (figure 3). Using the End TB Strategy’s target of 80% incidence reduction by 2030 as the reference, several countries came very close to reaching the 2030 target in this optimal scenario, including India (61.9%, 30.2%–83.5%), Brazil (56.5%, 48.2%–64.1%), Viet Nam (55.0%, 26.8%–75.9%), Pakistan (54.0%, 33.2%–73.8%), Bangladesh (52.8%, 24.3%–75.4%), Cambodia (51.4%, 32.2%–70.1%), China (50.9%, 34.5%–66.1%) (online supplemental appendix 4).

**Table 2:** Country-specific reduction in tuberculosis incidence and mortality compared with the current level scenario

	Continuing trend		Current level and zero hunger		Continuing trend and zero hunger	
	TB disease prevented (1000s)	TB deaths averted (1000s)	TB deaths averted (1000s)	TB deaths averted (1000s)	TB disease prevented (1000s)	TB deaths averted (1000s)
Bangladesh	377 (227–548)	108 (45–199)	1074 (336–1832)	342 (122–667)	1192 (510–1922)	367 (139–700)
Brazil	57 (38–82)	9 (4-16)	59 (15–122)	11 (3–24)	95 (51–159)	16 (7–31)
Cambodia	127 (79–175)	25 (8–46)	214 (94–335)	46 (15–89)	252 (138–368)	52 (17–97)
China	1589 (1079–2002)	155 (60–295)	1810 (532–3119)	203 (61–424)	2601 (1477–3717)	268 (100–525)
India	2090 (985–3458)	500 (54–1324)	8652 (2627–15983)	2222 (236–6150)	9209 (3316–16542)	2325 (248–6361)
Indonesia	1770 (1248–2335)	724 (428–1100)	2637 (1018–4330)	1259 (551–2131)	3370 (1910–4924)	1488 (782–2380)
Myanmar	385 (266–505)	88 (35–151)	445 (169–731)	118 (41–222)	607 (365–860)	147 (57–260)
Pakistan	736 (464–1026)	184 (56–348)	1084 (357–1874)	315 (87–642)	1406 (745–2152)	378 (114–738)
Philippines	421 (265–582)	79 (25–143)	769 (265–1298)	166 (49–327)	944 (472–1451)	192 (60–364)
Russia	108 (70–149)	21 (11–35)	90 (22–179)	24 (7–49)	170 (95–265)	38 (18–67)
Thailand	156 (98–221)	40 (16–76)	211 (57–390)	64 (19–137)	290 (148–463)	80 (30–160)
Viet Nam	209 (118–290)	31 (11–61)	437 (154–685)	68 (22–141)	483 (221–715)	73 (25–149)
**Total**	8025 (4937–11373)	1964 (753–3794)	17 482 (5657–30878)	4838 (1213–11003)	20 619 (9449–33538)	5424 (1597–11832)

TB, tuberculosis.

In sensitivity analyses, reductions in TB incidence in the current level and zero hunger scenario compared with the current level scenario were sensitive to parameters for the relative risk of TB incidence for those underweight compared with normal weight, and the transmission parameter (online supplemental appendix 5).

## Discussion

Compared with a scenario where mean BMI remained at 2015 levels, if past trends in mean BMI continued, the proportion of the population overweight and obese would increase and the proportion of underweight would decrease. Then by 2030, TB incidence and mortality would further decline by a cumulative 14.7% (12.7%–16.7%) and 15.6% (12.5%–19.2%), respectively. This would occur due to both a simultaneous decrease in the harmful effect of underweight and an increase in the protective effect (despite the possibility of diabetes) of being overweight and obesity. In comparison, achieving zero hunger by 2030, without an increase in overweight or obesity, would reduce incidence and mortality by 32.0% (20.0%–43.8%) and 37.3% (26.1%–49.6%), respectively. If past trends continued and zero hunger was also achieved, incidence and mortality would be reduced by 38.2% (27.0%–49.1%) and 42.4% (32.1%–53.5%), respectively, equivalent to preventing 20.6 million TB cases and averting 5.4 million TB deaths between 2015 and 2030.

We postulate that the large effect of zero hunger in some countries was attributed to a corresponding higher burden of underweight. Among the 12 countries, Bangladesh, India and Vietnam had the top 3 highest underweight prevalence in 2015. We found that the cumulative reduction of TB incidence in the zero hunger scenario was also the highest in these three countries. The reduction of TB incidence was found to be highly correlated with the prevalence of underweight (r=0.70, p=0.011). In the continuing trend scenario, however, those with currently rapidly increasing prevalence of overweight and obesity such as Myanmar and Cambodia, rather than those with a higher overall prevalence such as Brazil and the Russian Federation, were projected to have a more rapid decline in TB burden. While the combined scenario of continuing trend and zero hunger had the largest impact on TB, the net effect was mainly influenced by zero hunger in most countries (except for Brazil and the Russian Federation), with some additional impact due to continuing trend (figure 2). Results were qualitatively similar for TB incidence and mortality.

Previous work has demonstrated the impact of reducing undernutrition on TB in the Central Eastern states of India,^28^ demonstrating a similar overall reduction in incidence to our results (a 71% vs 68% reduction respectively from 2030 compared with 2011). A recent modelling analysis of countries in the South-East Asia Region, based on the results from the RATIONS trial, revealed that nutritional interventions could substantially impact TB epidemiology.^17^ It was found that population-based interventions would have a much greater effect than interventions targeting only the close contacts of TB patients. The results from our analysis of reducing undernutrition (the zero hunger scenario) were in line with the findings from the previous literature. The unique contribution of the present analysis is the consideration of the impact of overnutrition on TB incidence, incorporating changes in the prevalence of DM resulting from increasing obesity. Using robust BMI trend estimates and methods to account for the interrelation between undernutrition and overnutrition,^14^ our analysis explored the impact of global nutritional transition (the continuing trend scenario) on TB in high burden countries. Our results show how population nutrition level could affect TB more widely, including in countries where undernutrition is a less critical driver of TB burden.

When estimating the effect of overnutrition on the risk of TB, we included diabetes as a mediator between overweight/obesity and TB. This was done by limiting the BMI and TB associations to cohort studies which provided estimates of the total effect of BMI on TB (ie, not adjusting for diabetes in the analysis, Supplemental Figure the DAG). Our previous studies of two large population-based cohorts revealed that the diabetes-mediated (indirect) effect would result in a positive association between BMI and TB risk, but the direct effect of BMI on TB was protective and much stronger than the diabetes-mediated effect, making an overall net protective effect of BMI on TB risk. Although we accounted for the impact of diabetes on the BMI and TB association through a mediation analysis framework, we did not explicitly account for the co-occurrence of overnutrition and diabetes which may arise from common causes (such as lifestyle and dietary changes). Therefore, we could not completely rule out the possibility that the co-occurrence of diabetes along with overnutrition (above and beyond the mediation effect) might undermine (at least partially) the beneficial effect of increasing BMI on TB epidemiology. Additionally, the association between BMI and diabetes risk varied considerably across populations and ethnic groups. Our model parameters were derived from cohort studies conducted in the USA and Taiwan and may not fully capture these differences. Future modelling studies that draw on the historical trajectories of the correlation between BMI increase and diabetes increase in different settings would help to better quantify the complex interplay between BMI, diabetes and TB trajectory.

One limitation of the present analysis is the lack of consideration of the effect of changing TB programmes, which could reduce the overall burden of TB and hence the potential for nutritional trends to affect TB. Another limitation is the use of case fatality rate estimates where the nutritional status (eg, low BMI) was assessed at the time of TB diagnosis. BMI at the time of TB diagnosis may be affected by disease-related weight loss and served more as a mark of disease severity than of pre-existing nutritional status. This constraint of available data could have overestimated the case fatality rate among underweight individuals and the contribution of undernutrition to TB mortality in our model. Additionally, nutritional targets and representation of BMI in our model lack granularity on age and sex; both TB^29^ and nutritional status^14 30^ are highly gendered and age-varying. For example, reaching Zero Hunger could have a greater effect on men (who bear a greater burden of TB,^29^ and in many settings have a lower BMI).^31^ Additionally, we assumed zero hunger led to an increase in normal weight rather than overweight or obese, which could have provided a protective effect against TB. As such, our results may be conservative. Lastly, population nutritional level was assumed to be an exogenous factor, which would not be affected by changing TB epidemiology. If the benefits of nutritional transition on TB control would lead to further improvement in population nutrition, our estimates of the effect of nutritional transition would be conservative.

Nutrition is a critical determinant of TB, providing a protective effect at high BMI despite the risks of diabetes, and incurring an increased risk of TB disease at low BMI. Future trends in nutrition are therefore likely to have a significant impact on TB epidemiology. Our analysis of 12 high TB burden countries revealed that a substantial burden of TB incidence and mortality could be averted through a decrease in population-level undernutrition and an increase in BMI. In several countries under investigation, the combined effect of increased BMI and ending hunger could lead to a 50%–60% reduction in TB incidence by 2030. This magnitude of reduction would substantially contribute to achieving the End TB Strategy’s target of an 80% reduction in TB incidence by 2030. Our results highlight the importance of the Sustainable Development Goals to end hunger on infectious disease control, and the benefits of an integrated approach to health for all. Nutrition transitions and interventions to end hunger could have a major impact on the future epidemiology of TB in high burden countries, and national TB programmes should coordinate with non-communicable disease sectors when setting targets for TB.

## Supplementary material

10.1136/bmjgh-2024-018839 online supplemental file 1

10.1136/bmjgh-2024-018839 online supplemental file 2

10.1136/bmjgh-2024-018839 online supplemental file 3

## Data Availability

Data are available on reasonable request.
