# Supplementary material for: Estimating the impact of nutritional transition and ending hunger on tuberculosis in 12 high-burden countries: a model-based scenario analysis
Source: BMJ Glob Health. 2025 Dec 25;10(12):e018839. doi: 10.1136/bmjgh-2024-018839 (PMC12742055; doi:10.1136/bmjgh-2024-018839)
Supplement: online supplemental file 1 [file bmjgh-10-12-s001.docx]

**Supplemental Material**

[**Appendix 1. Model details** 2](#_Toc172553015)

[**1.1 Model equations** 2](#_Toc172553016)

[**1.2** **Model calibration** 3](#_Toc172553017)

[**Appendix 2. Country specific parameters** 5](#_Toc172553018)

[**2.1** **The effect of TB control program** 5](#_Toc172553019)

[**2.2** **Incidence fitting** 7](#_Toc172553020)

[**2.3** **Prior distribution versus Posterior distribution** 8](#_Toc172553021)

[**Appendix 3. Crosswalk- mean BMI to categorical BMI prevalence** 15](#_Toc172553022)

[**Appendix 4. Country specific results** 16](#_Toc172553023)

[**Appendix 5. Sensitivity** 27](#_Toc172553024)

**Appendix 1****. Model details**

**1.1 Model equations**

For each country, we constructed a dynamic compartmental model incorporating the natural history of tuberculosis (TB), stratified by body mass index (BMI) into four categories, underweight (<18.5 kg/m^2^), normal (>=18.5 and <25 kg/m^2^), overweight (>=25 and <30 kg/m^2^) and obesity (>=30 kg/m^2^). Individuals were grouped accordingly, underweight as Group 1, normal weight as Group 2, overweight as Group 3, and obese as Group 4. In the model, all individuals without TB face the general mortality rate (denoted as $u)$, which varies by BMI levels. The structure of the model remains consistent across BMI categories. Each BMI sub-model ran separately but was connected by a common force of infection (driven by all prevalent TB cases in the population) assuming homogeneous mixing. Epidemiological indicators such as incidence and prevalence are calculated as weighted averages based on the distribution of BMI categories in the population.

**1.1.1 Susceptible (S_i_)**

All individuals begin in the susceptible compartment and can become infected with TB at a rate determined by the annual risk of infection (ARI).

$${D\left( S_{i} \right)}/{dt}=Birth-DrFun*{uRR}_{i}*S_{i}-ari*S_{i}$$

$$ari=\beta*sum(I_{i}*{probBMI}_{i})$$

$(where i represents the BMI category, i=1:underweight, i=2:normal BMI, i=3:overweight, i=4 obesity$)

**1.1.2 Fast latent infection (**$\boldsymbol{Lf}_{\boldsymbol{i}}$**)**

Individuals in the fast latent infection compartment have a higher probability of progressing to active disease through primary progression ($p)$ within the initial years following infection. Alternatively, they may transition to the slow latent infection state at rate $k$.

$${d\left( {Lf}_{i} \right)}/{dt}=ari*S_{i}-(u*{uRR}_{i}+k+p*{pRR}_{i})*{Lf}_{i}$$

**1.1.3 Fast latent reinfection (**$\boldsymbol{Lf}_{\mathbf{i}}\boldsymbol{\_}\boldsymbol{re}$**)**

Individuals who have recovered or are in the slow latent infection status may be reinfected. Upon reinfection, they enter the fast latent reinfection compartment, from which they may either progress to active disease at a reduced primary progression rate $(jm)$ or transition to the slow latent infection state ($k)$.

$${d\left( {Lf}_{i}\_re \right)}/{dt}=\beta*I*({Ls}_{1}+R_{1})-(u*{uRR}_{i}+k+{jm}_{i})*{Lf}_{i}\_re-r_{i}\_Lfre$$

$({jm}_{i}=p*{RR}_{i}*\left( 1-im \right) ; im$: partial immunity)

**1.1.4 Slow latent infection (**$\boldsymbol{Ls}_{\mathbf{i}}$**)**

Individuals in the slow latent infection compartment may either be reinfected or develop active disease through endogenous reactivation at rate ($ra)$.

$${d\left( {Ls}_{i} \right)}/{dt}=k*\left( {Lf}_{i}+{{Lf}_{i}}_{re} \right)+nc*I_{i}-\left( u*{uRR}_{i}+ra*ra{RR}_{i} \right)*{Ls}_{i}-\beta*I*{Ls}_{i}-r_{i}\_Ls$$

**1.1.5 Active disease (**$\boldsymbol{I}_{\mathbf{i}}$**)**

Individuals in the active disease compartment may exit through one of the following pathways: natural cure ($nc$), successful treatment under the DOTs program ($d*ts)$ ,or TB-related mortality ($utb)$. Here, *d* denotes the case detection rate, and *ts* is the treatment success rate.

$${d\left( I_{i} \right)}/{dt}=ra*{raRR}_{i}*{Ls}_{i}+e*{eRR}_{i}*R_{i}+{jm}_{i}*{Lf}_{i}\_re+p*p{RR}_{i}*{Lf}_{i}-\left( utb*{utbRR}_{i}+nc \right)*I_{i}-d*ts*{Inc}_{i}-r_{i}\_I$$

**1.1.6 Recovered (**$\boldsymbol{R}_{\boldsymbol{i}}$**)**

Individuals who have recovered from TB may be reinfected or progress to active disease again through relapse at rate ($e)$.

$${d\left( R_{i} \right)}/{dt}=d*ts*{Inc}_{i}-\left( e*{eRR}_{i}+u*u{RR}_{i} \right)*R_{i}-\beta*I*R_{i}-r_{i}\_R$$

- 1. **Model calibration**

We calibrated each country-specific model to WHO estimates of TB incidence from 2006 to 2015 using an Approximated Bayesian Computation (ABC) rejection algorithm.[1] First, the distance metric was defined as the Euclidean distance between the simulated incidence and from the corresponding WHO country-specific estimates over the calibration period. A tolerance threshold was then defined as the top 5% of simulations with the smallest distances (i.e., 5% quantile of the distances from 100 preliminary runs). Parameter sets resulting in distances below this threshold were accepted. A total of 10,000 posterior parameter sets were collected through this rejection sampling process and used to estimate the impact of BMI on the future TB epidemic. Model simulations and statistical analyses were conducted using R version 3.3.3 (The R Foundation for Statistical Computing).

**
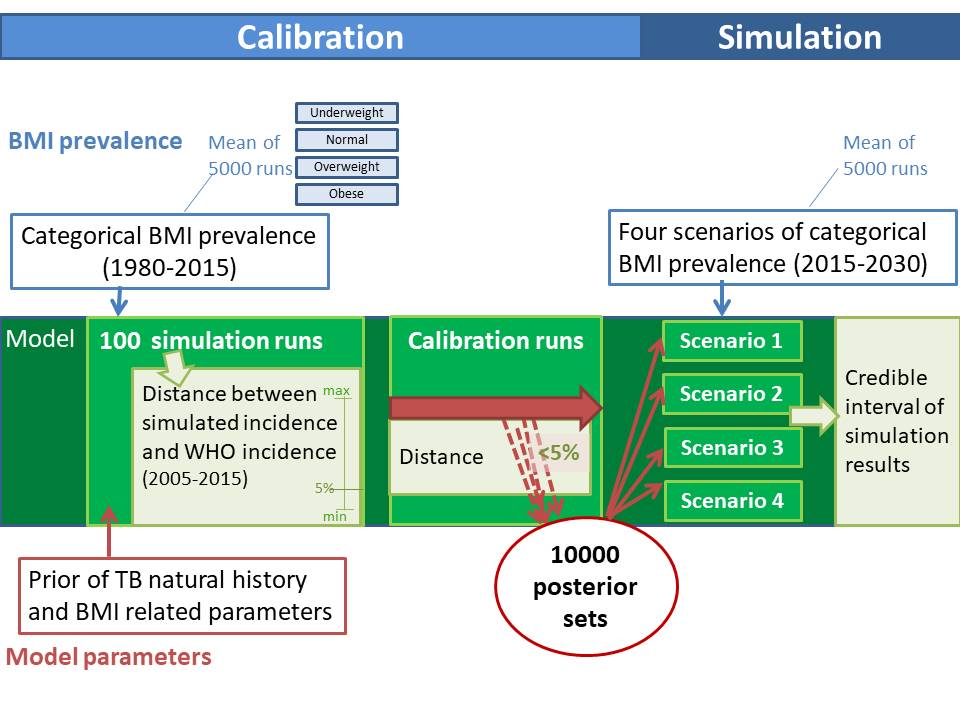
**

**Appendix 2. Country specific parameters**

- 1. **The effect of TB control program**

The impact of TB control programs was incorporated into the model through the inclusion of case detection rate and treatment success rate parameters. Due to limited availability of directly observed case detection rates, we fitted a logistic growth curve to the WHO’s 2014 estimates for each country. Treatment success rates were directly fitted to WHO estimates. Population dynamic was modeled by fitting growth trends to estimates from the United Nation Population Division (UNPD) [2]. The modeled population included only adults aged 15 years and older, consistent with WHO definition. Each country-specific model was calibrated to WHO TB incidence estimates from 2006 to 2015 [3] using the Bayesian melding approach [1]. Prior distributions for input parameters were listed in Table 2, and further details on the model structure and calibration procedures were included in Appendix 1.

- - **Case detection rate (%)**


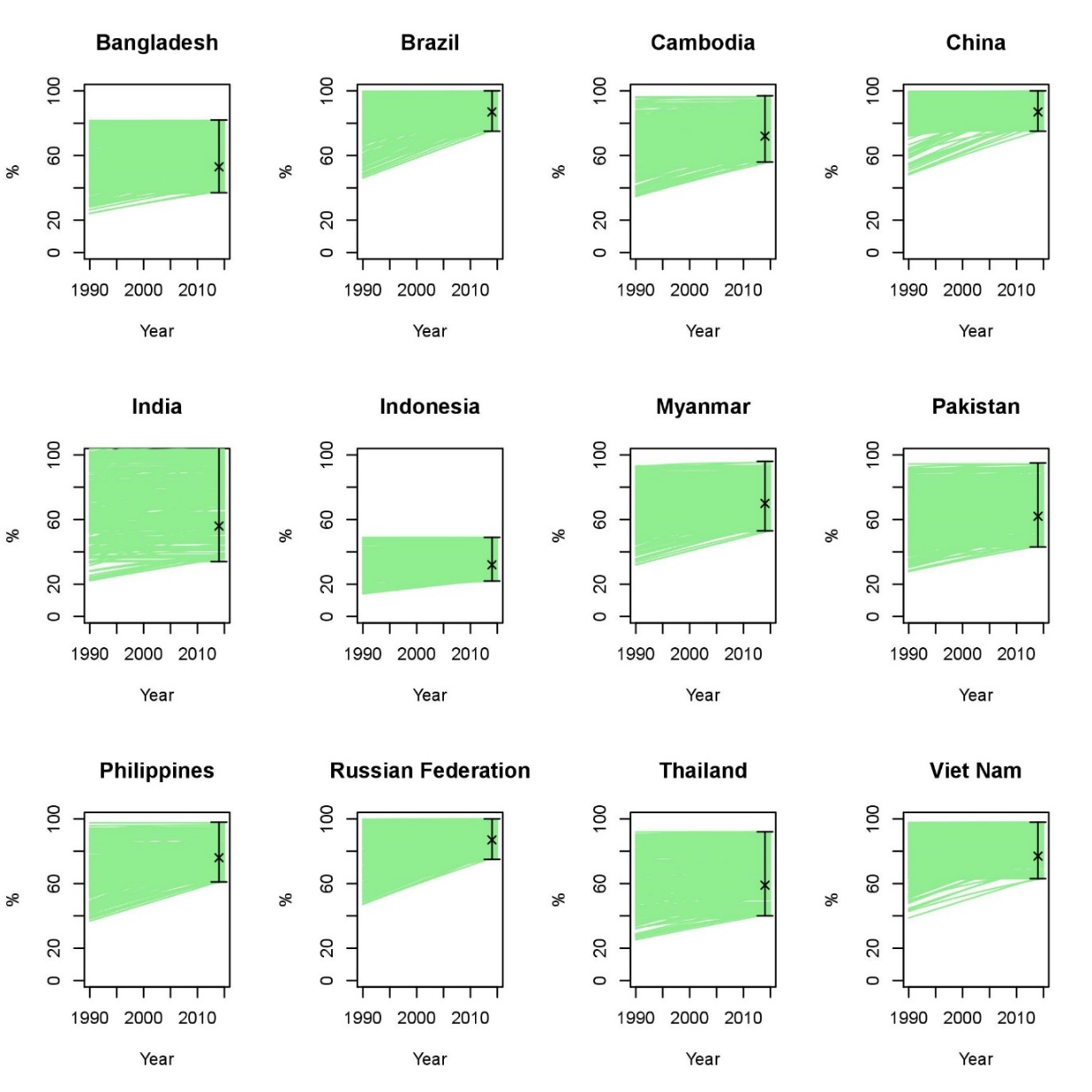


- - **Cure rate**


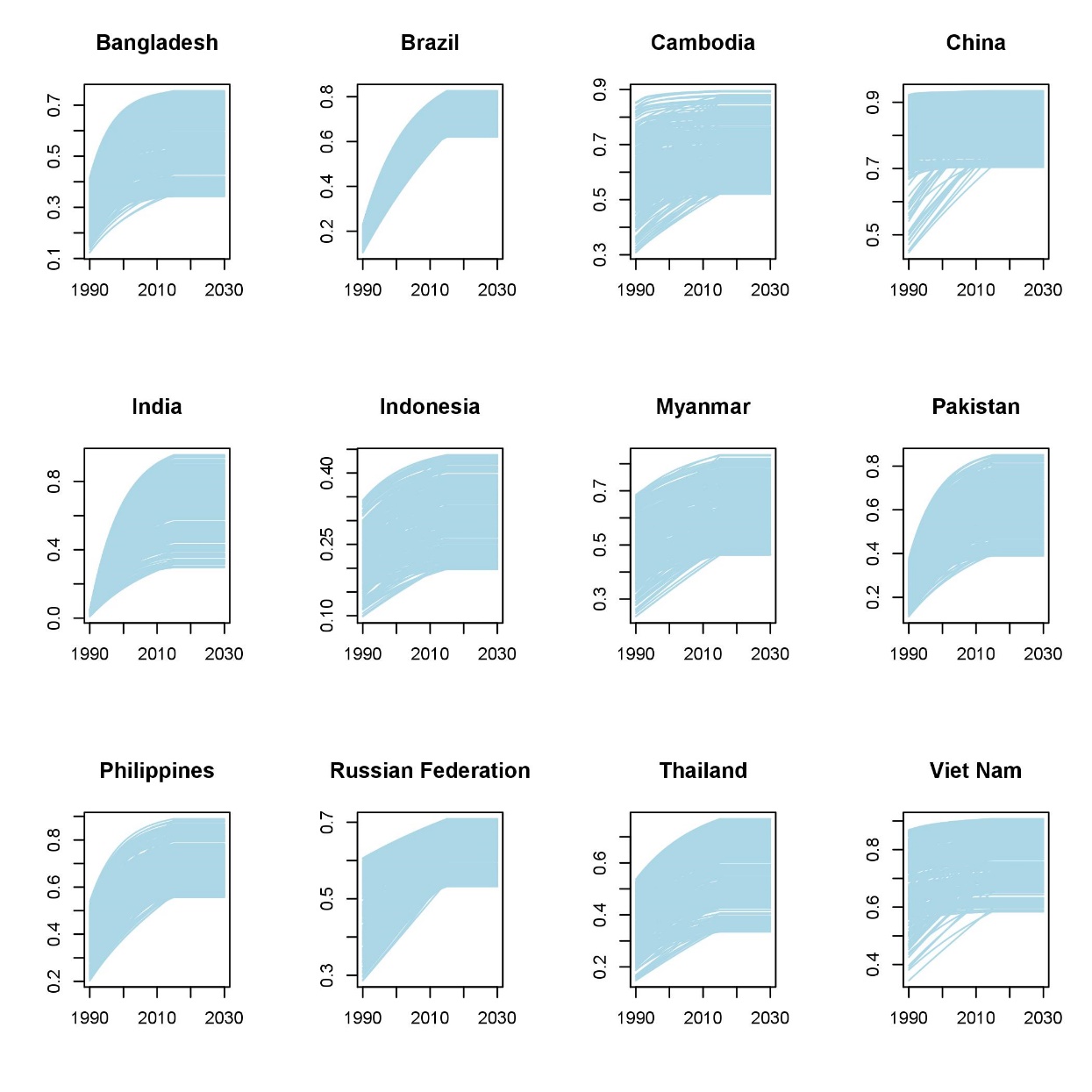


* **Cure rate = Case detection rate (cd)* Treatment success rate (ts)**

- 1. **Incidence fitting**


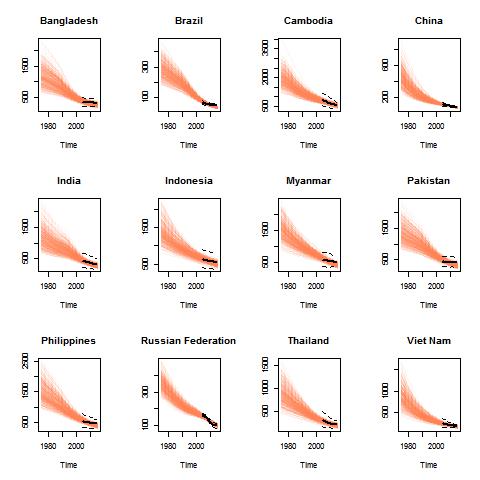


- 1. **Prior distribution versus Posterior distribution**


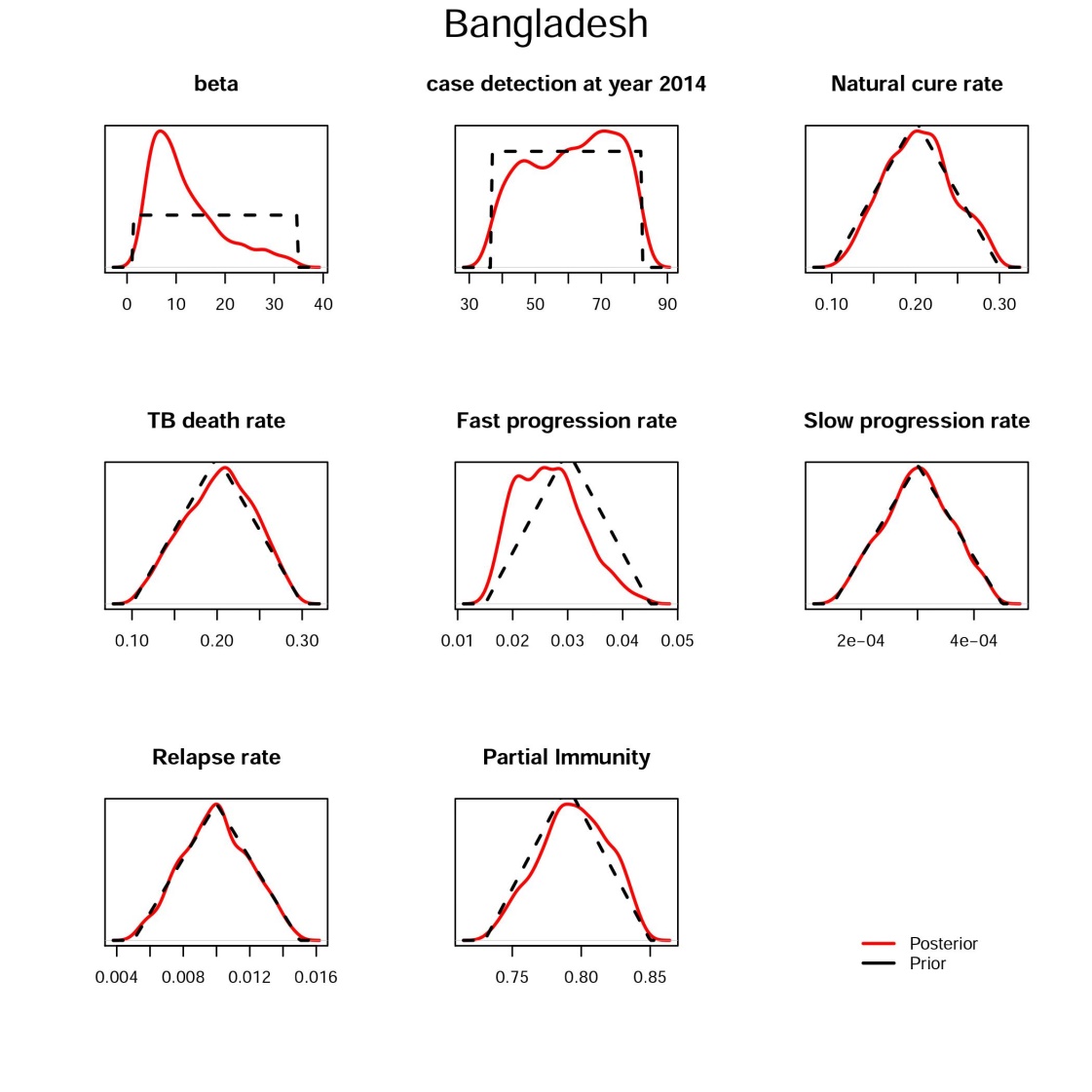

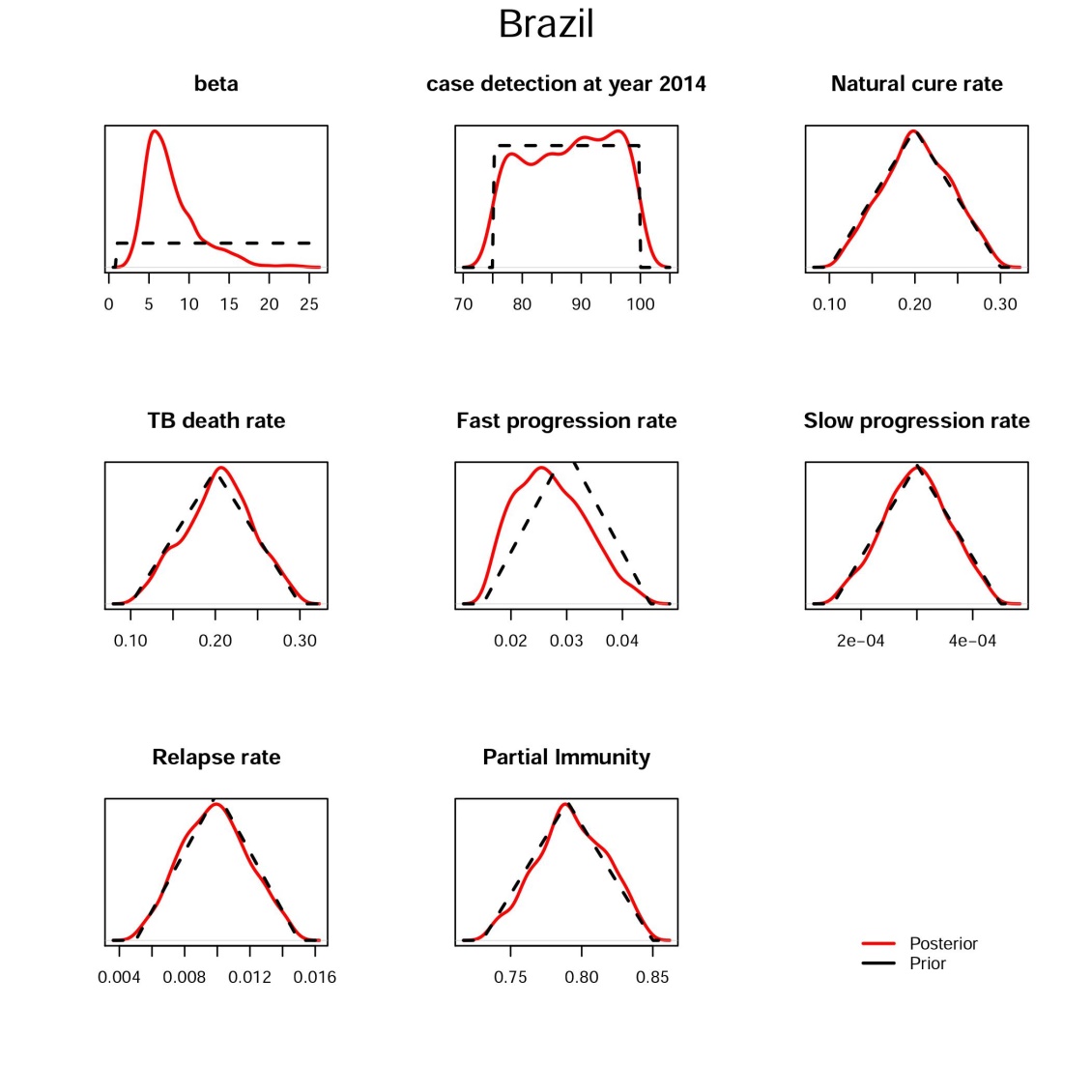

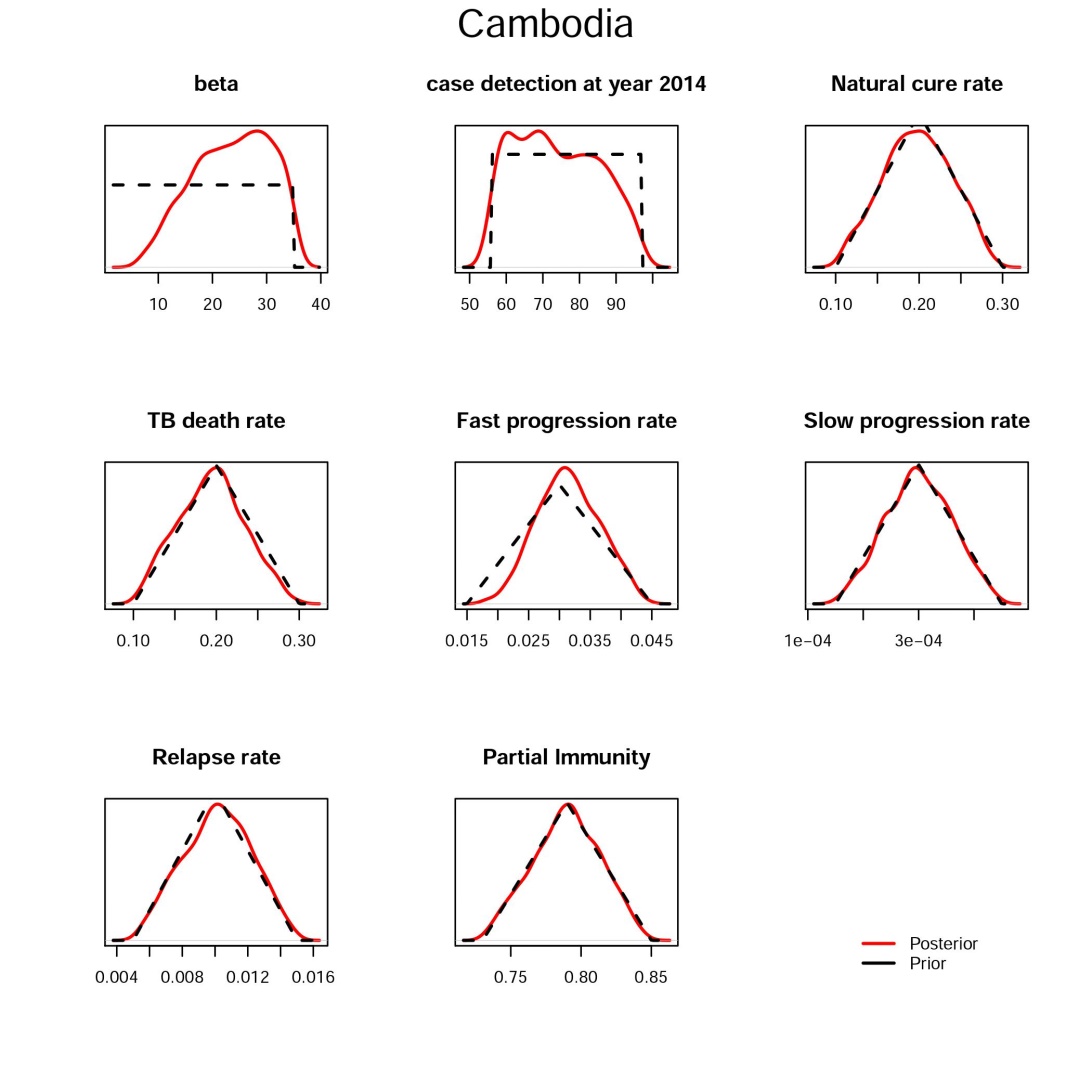

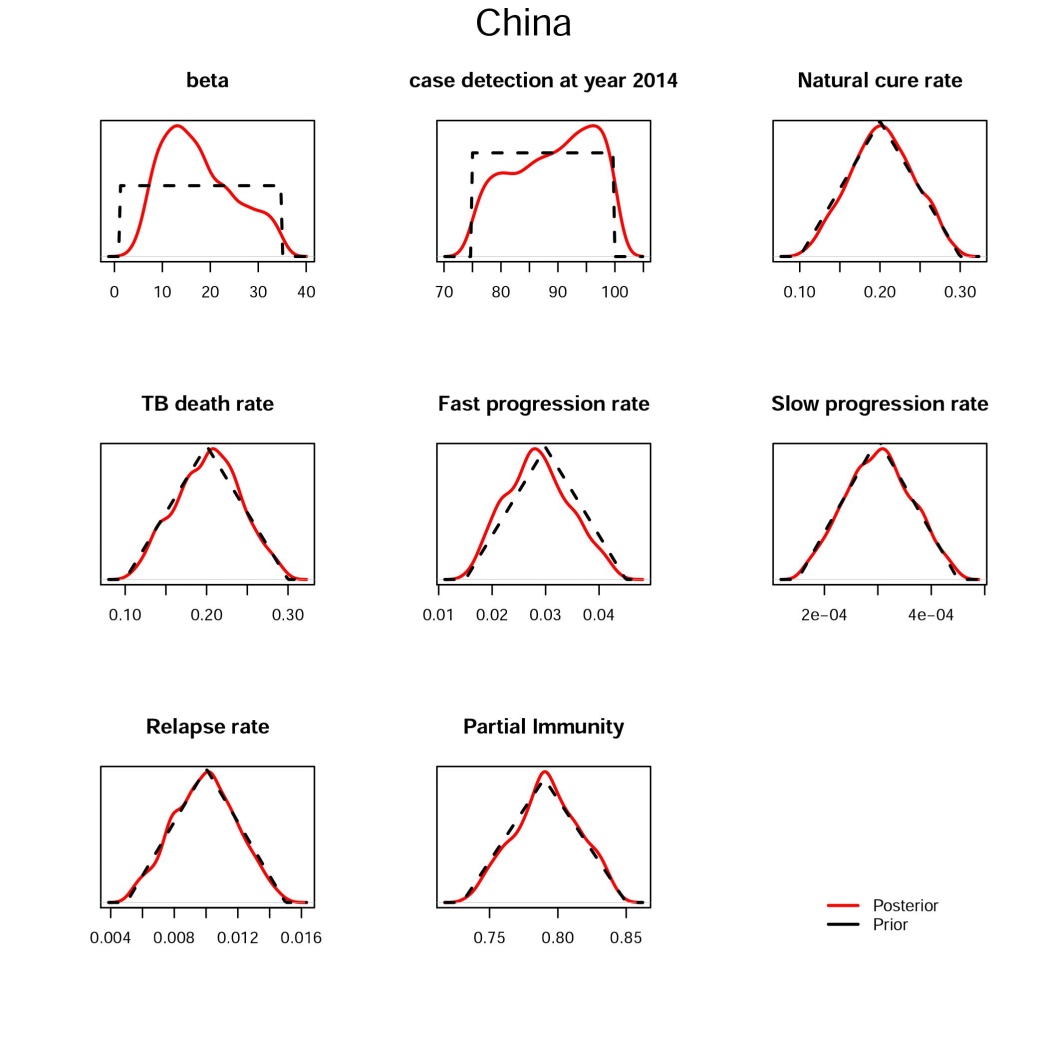

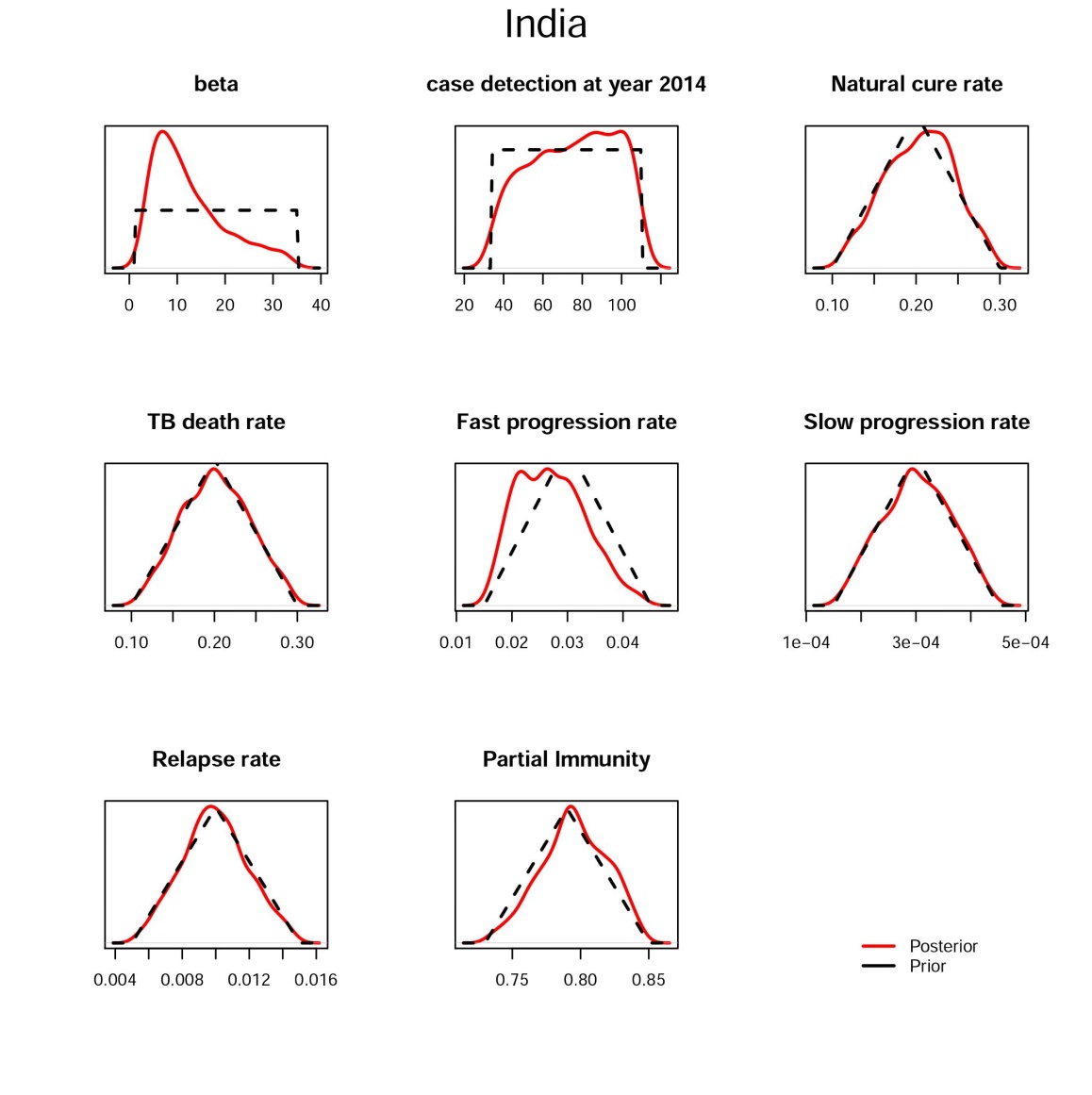

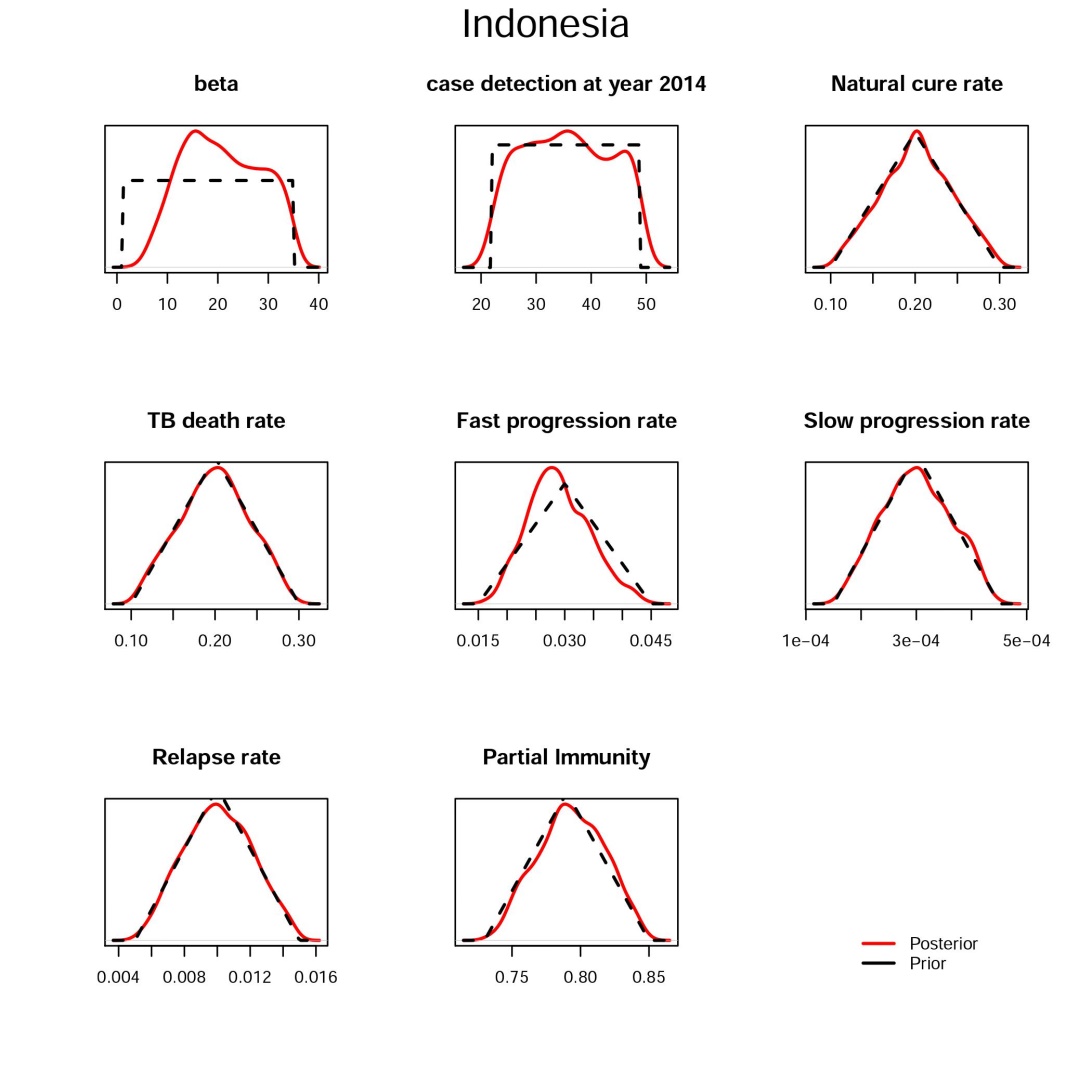

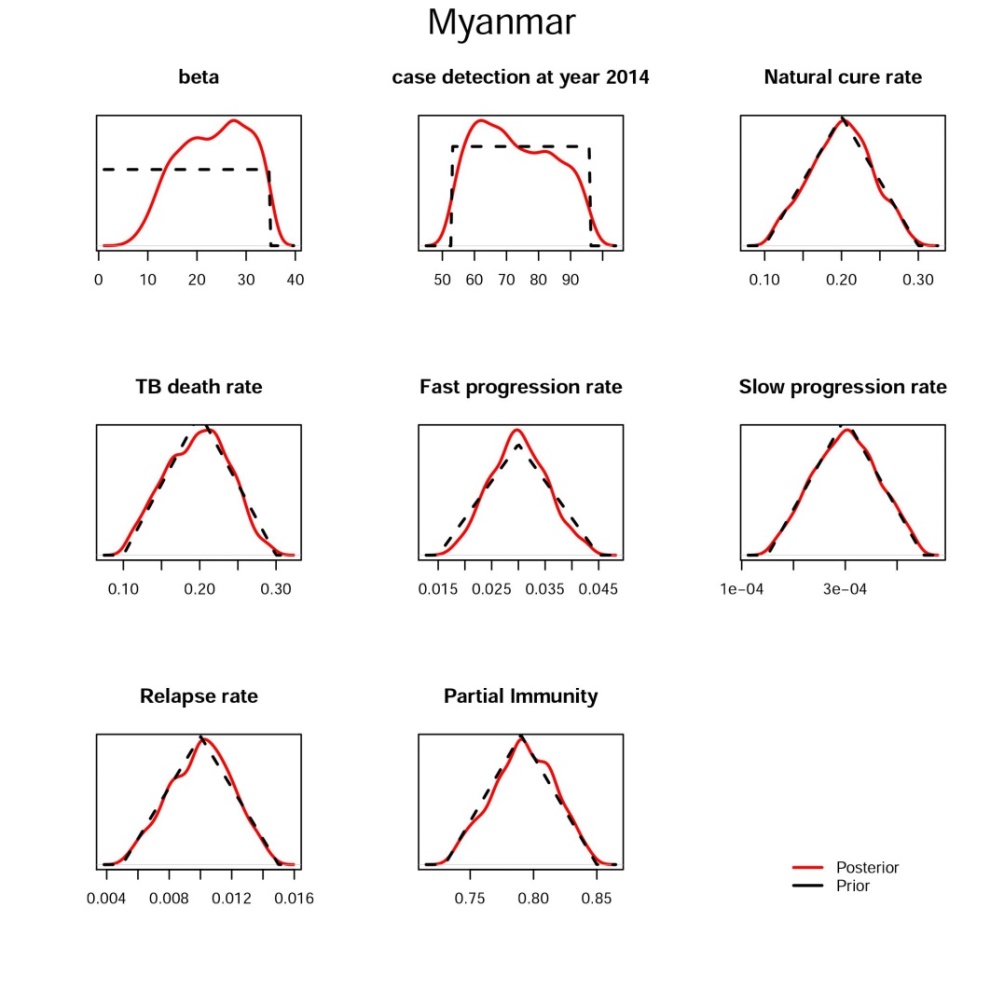


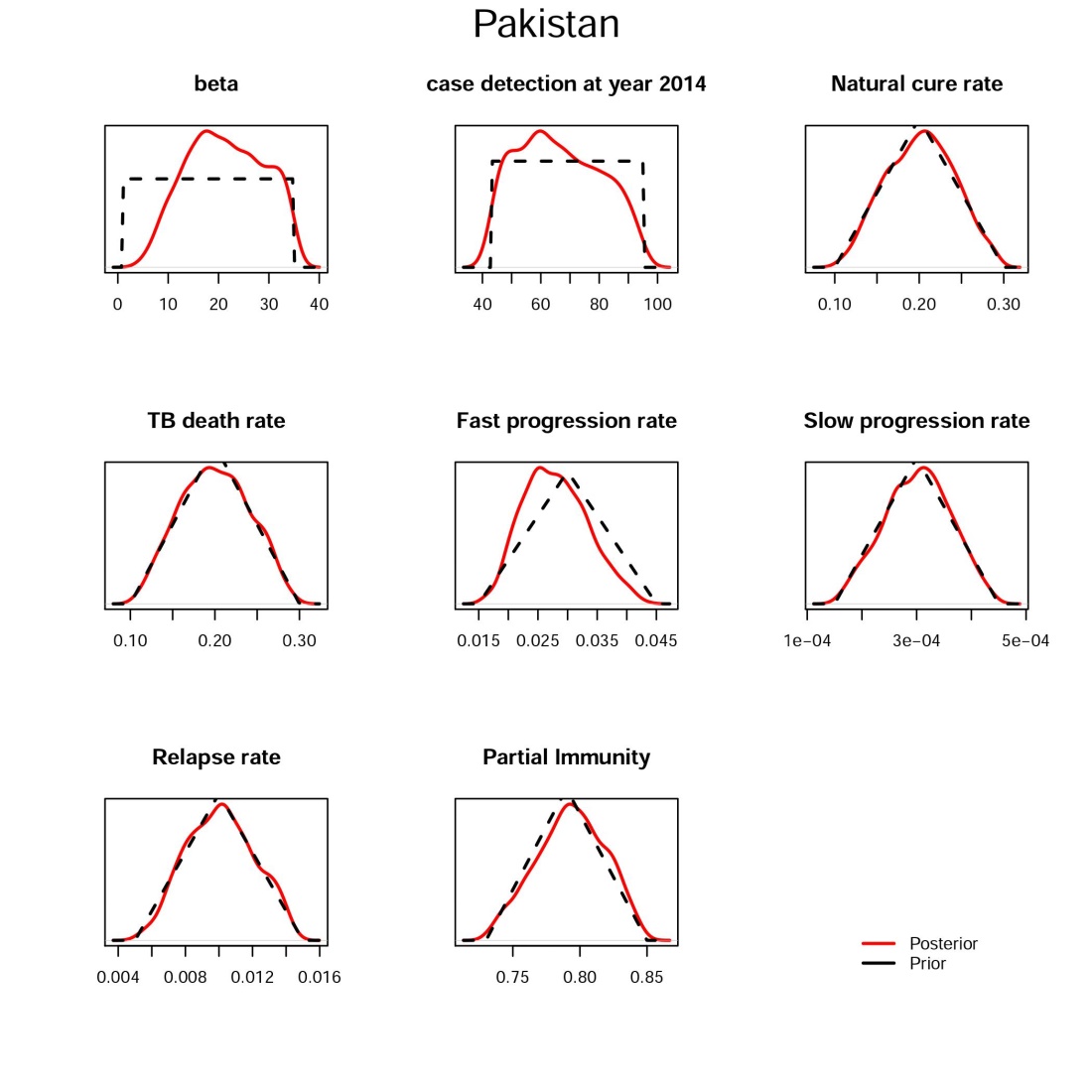

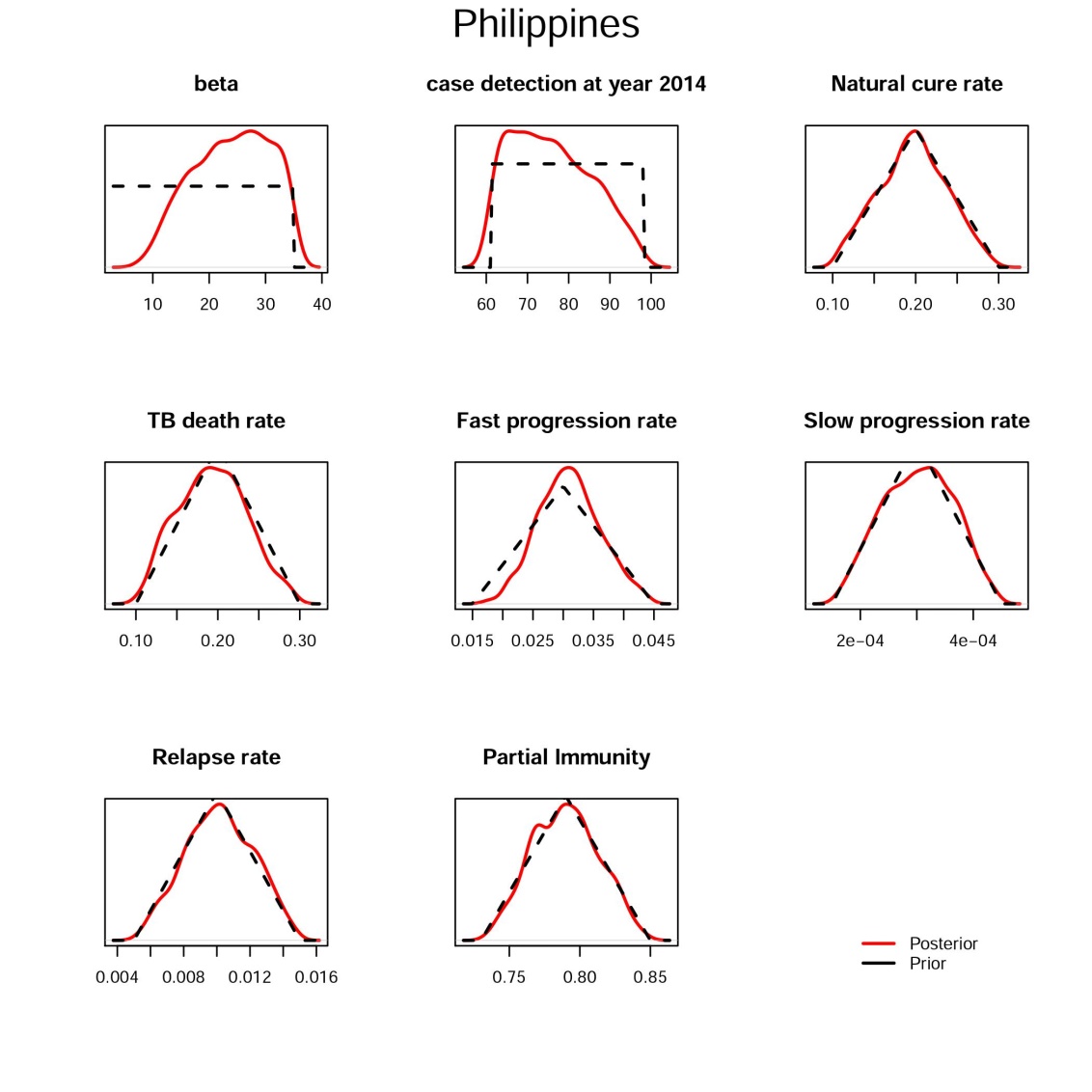

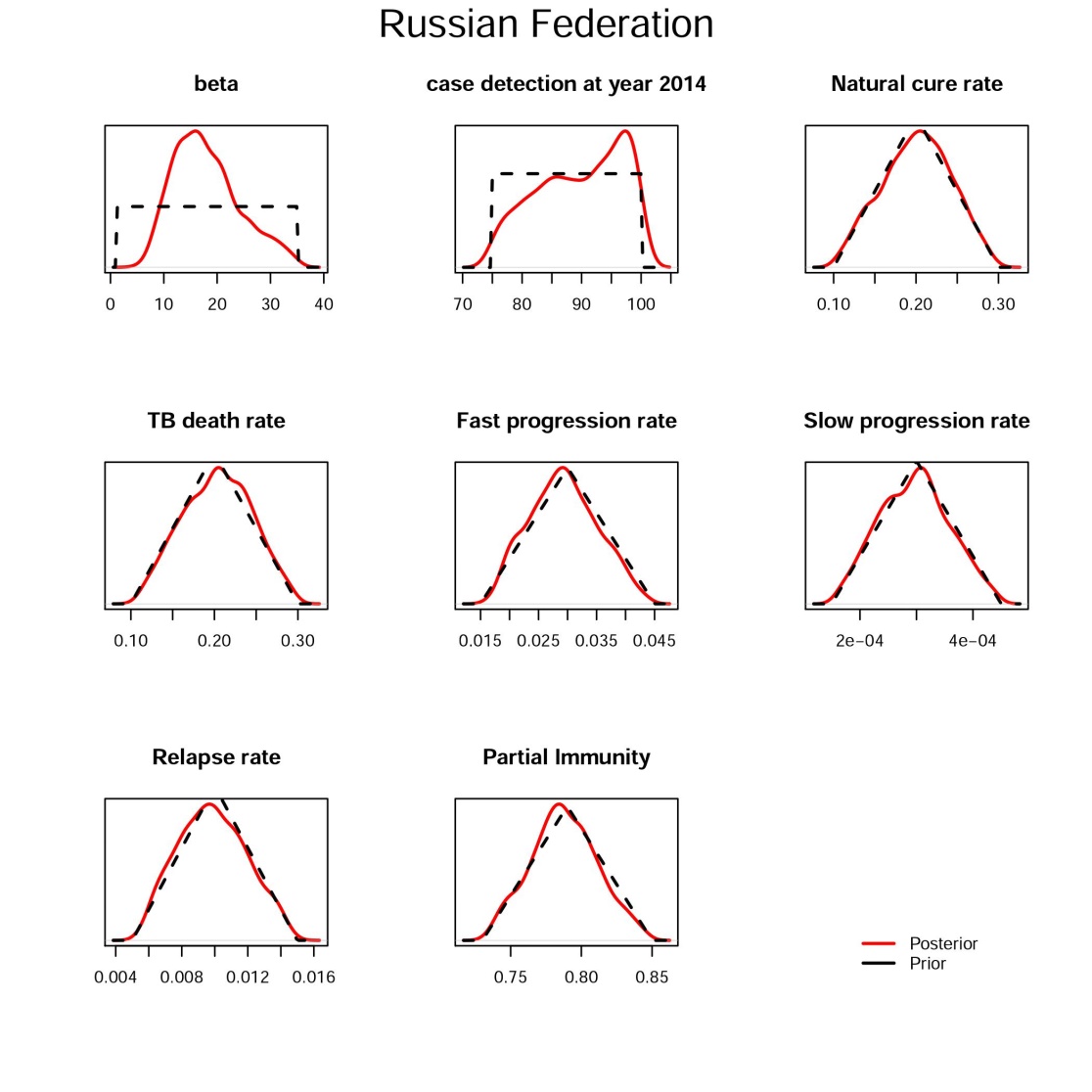

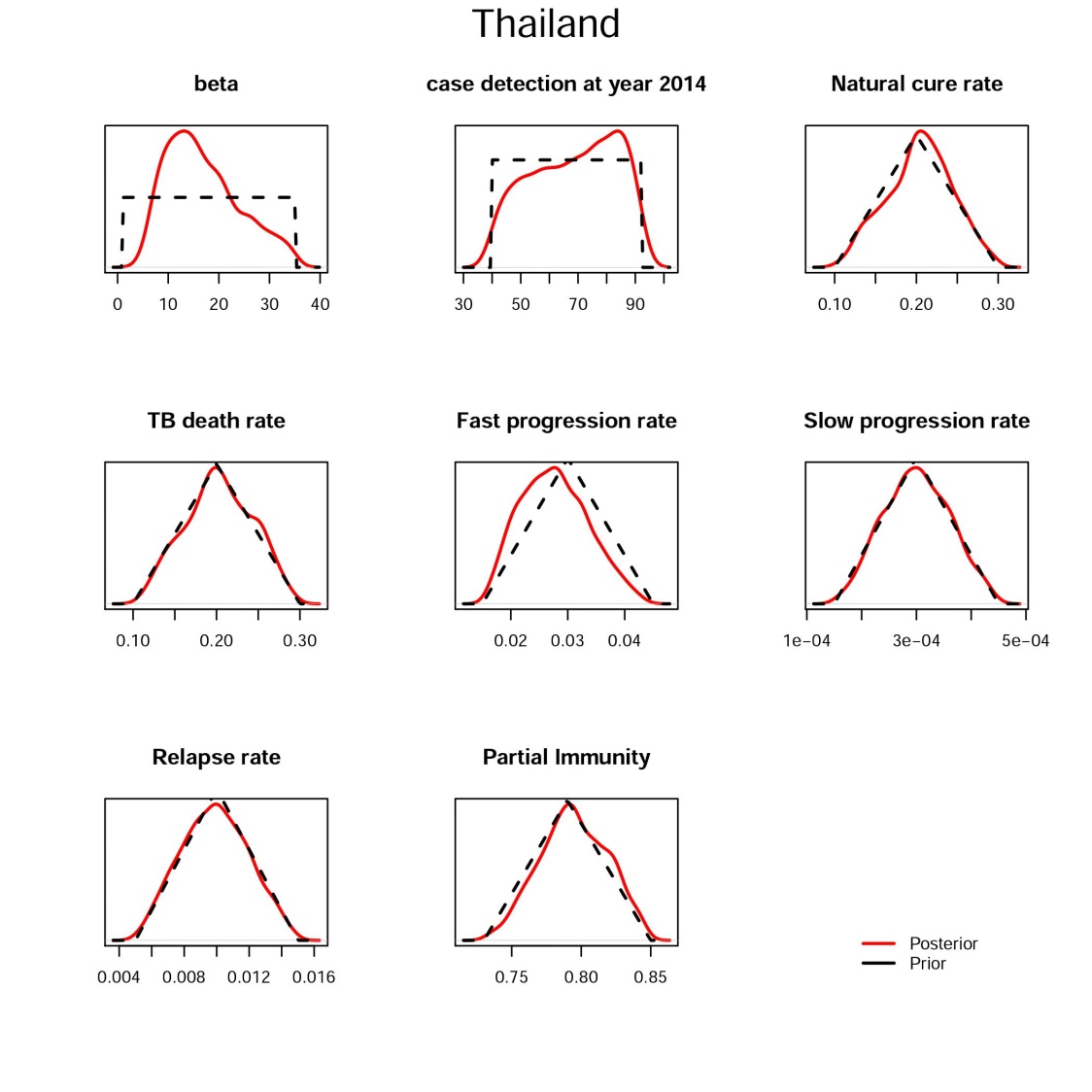

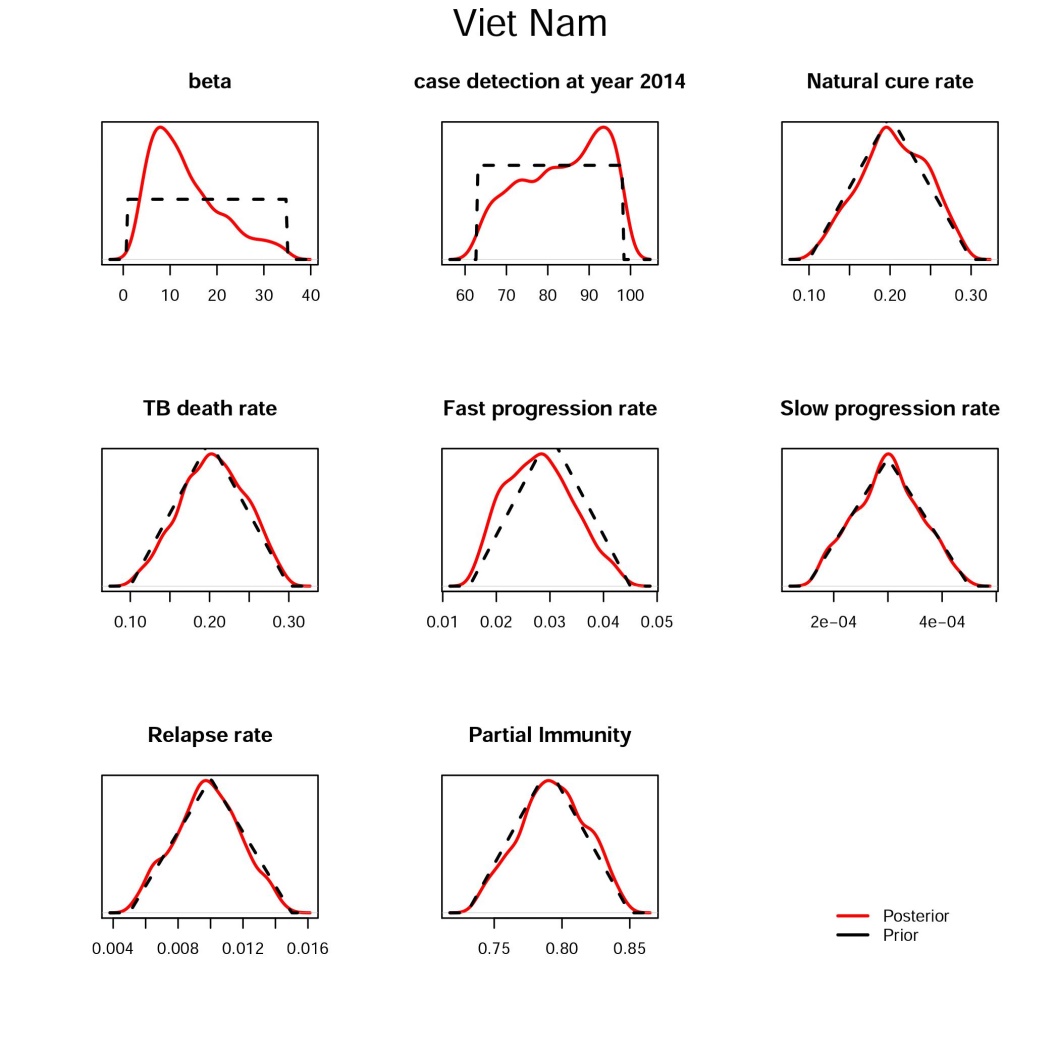


**Appendix 3. Crosswalk- mean BMI to categorical BMI prevalence**

Estimates of mean BMI were obtained from an updated systematic review [4], stratified by sex and 15 age groups. For each country and year, 5000 draws of mean BMI estimates were generated. Four scenarios were defined based on the projected trends in mean BMI for each age and sex group. To convert mean BMI to categorical BMI prevalence, we applied the cross-walk developed in the referenced review [4], This model used mean BMI, mean age, sex, study mid-year, and GDP as significant covariates. The trend of GDP was sourced from WHO estimates. The resulting categorical BMI prevalence for each age-sex group was weighted by the national population distribution to reflect country-level estimates. The final model input of categorical BMI prevalence was derived from taking the mean of the 5000 draws from the cross-walk conversion.


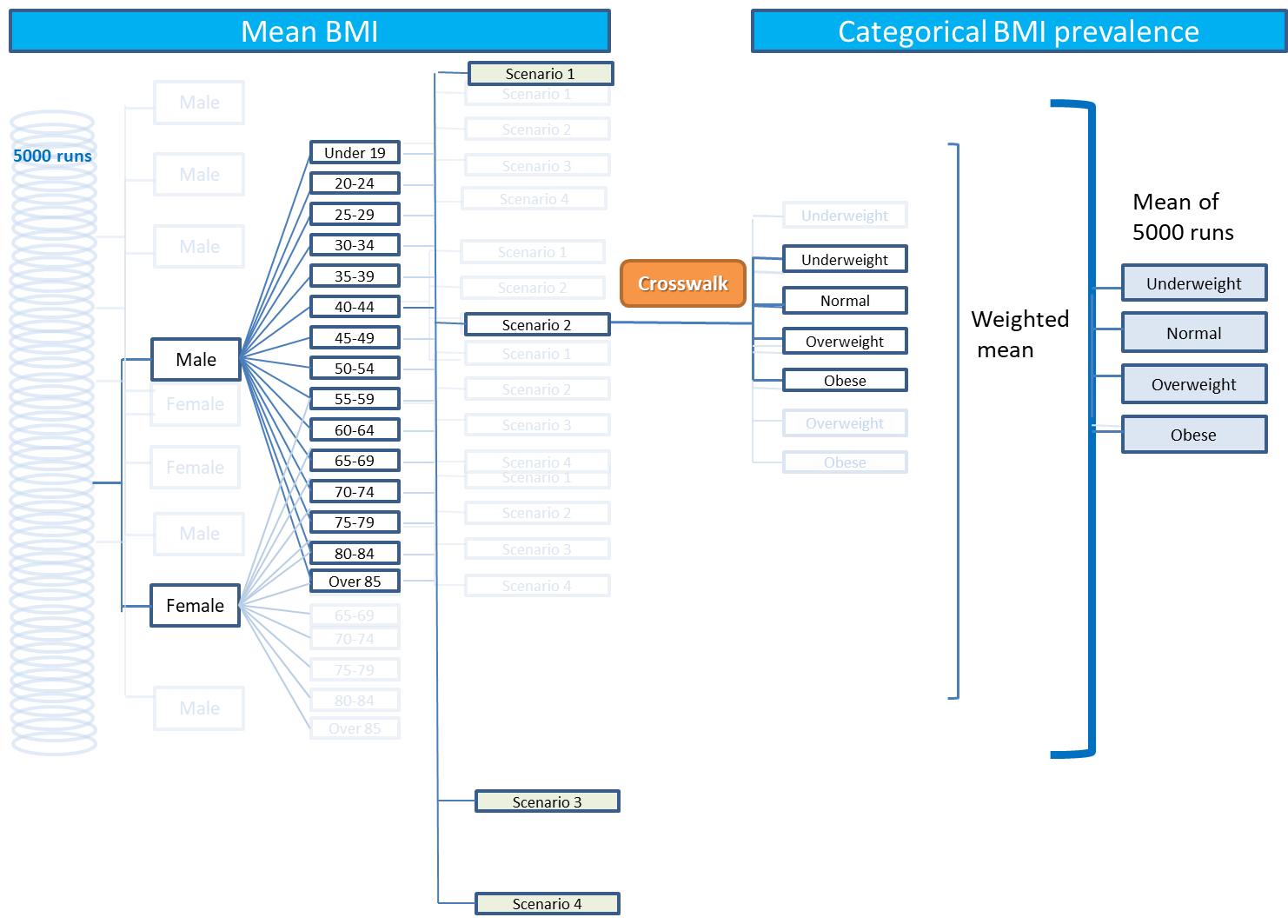


**Appendix 4. Country specific results**

- Categorical BMI prevalence projection
- TB incidence rate
- TB mortality rate
- Reduction of TB incidence
- Reduction of TB mortality


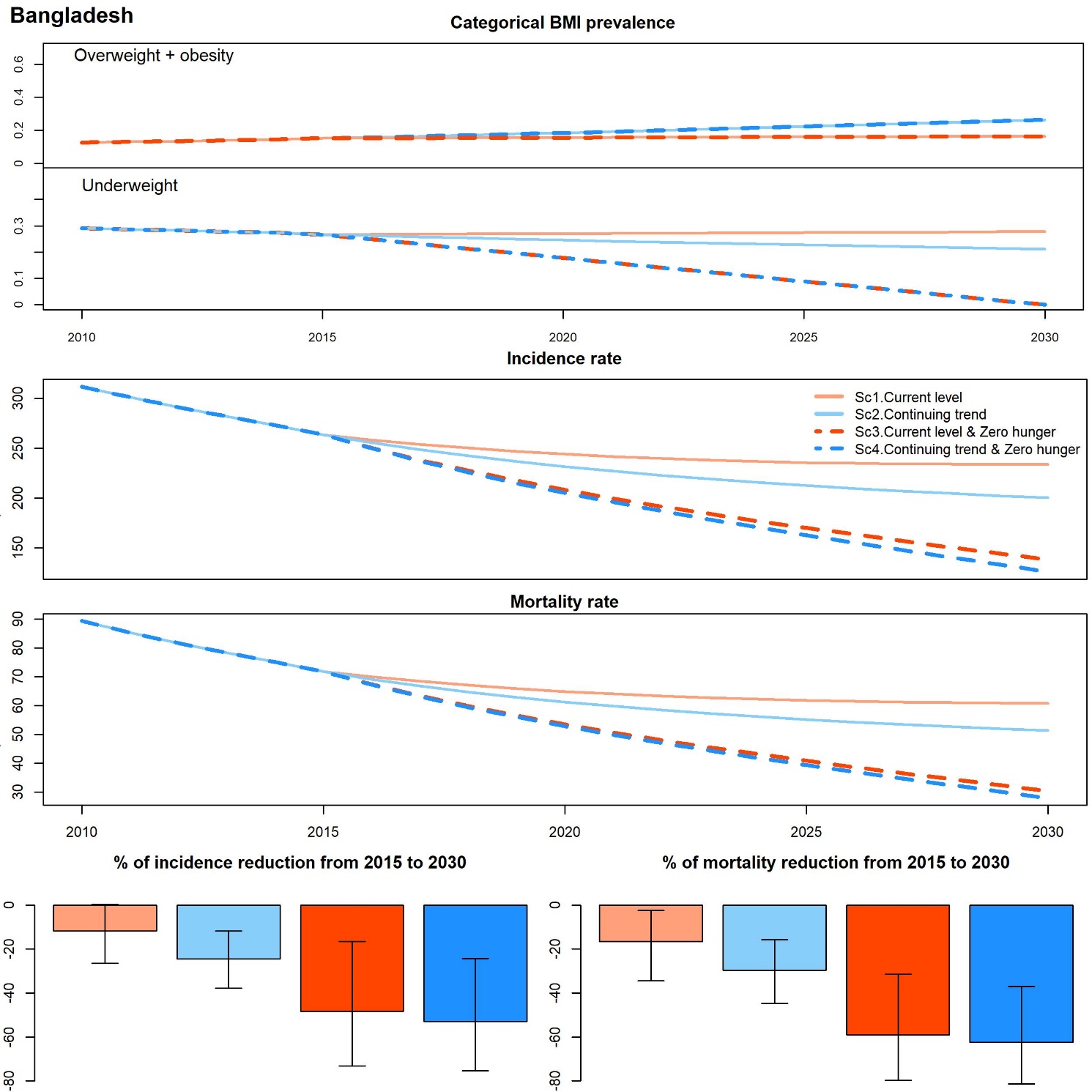


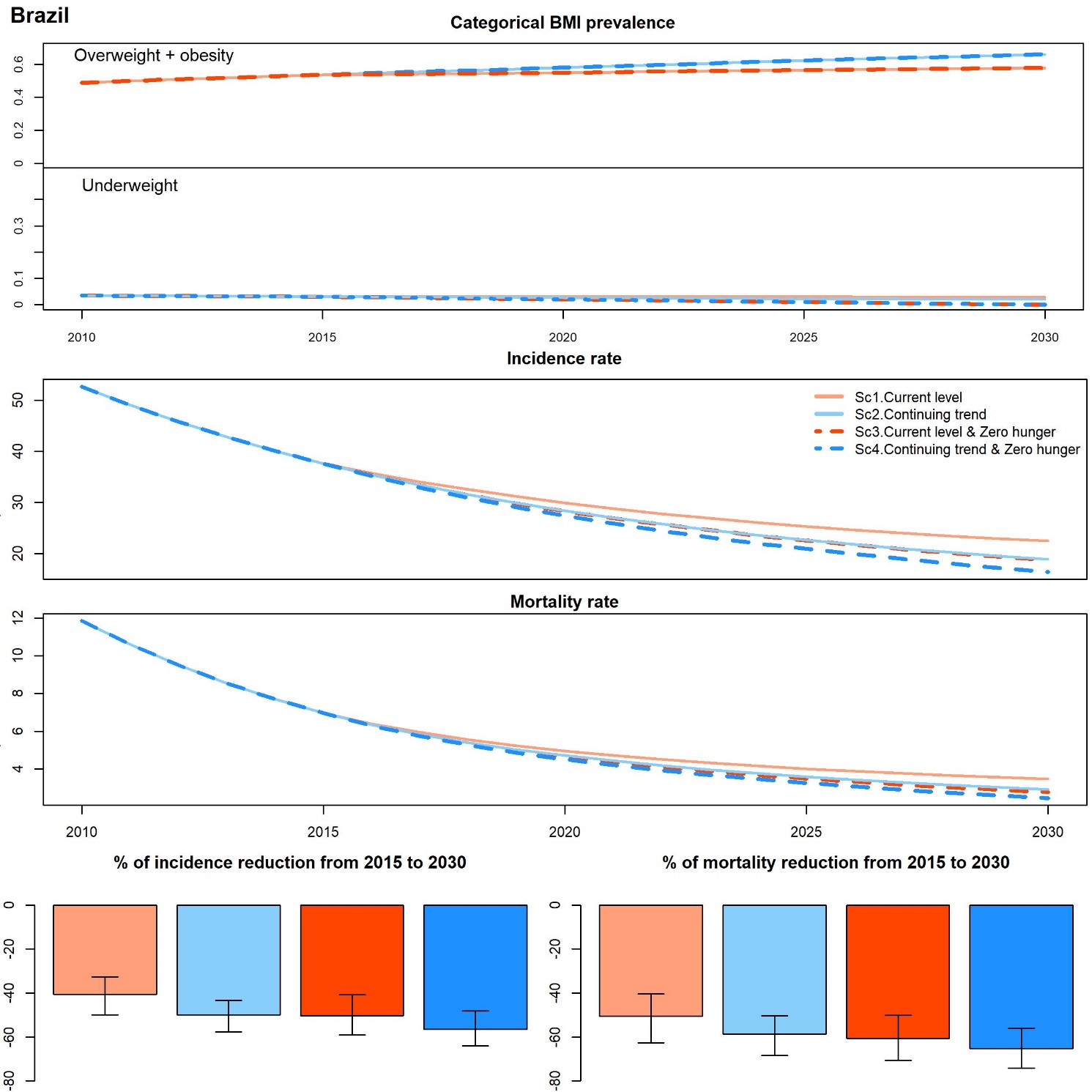


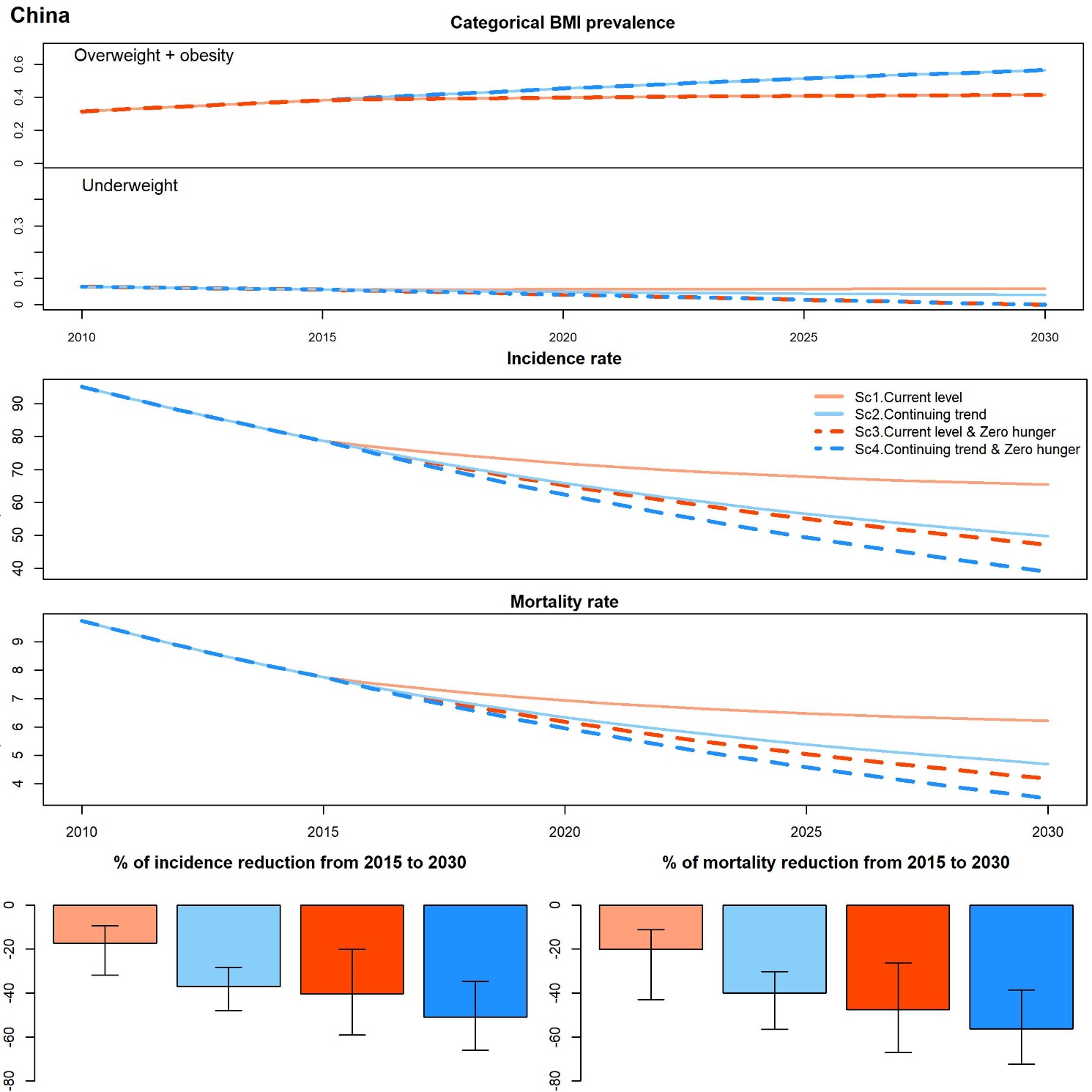


**
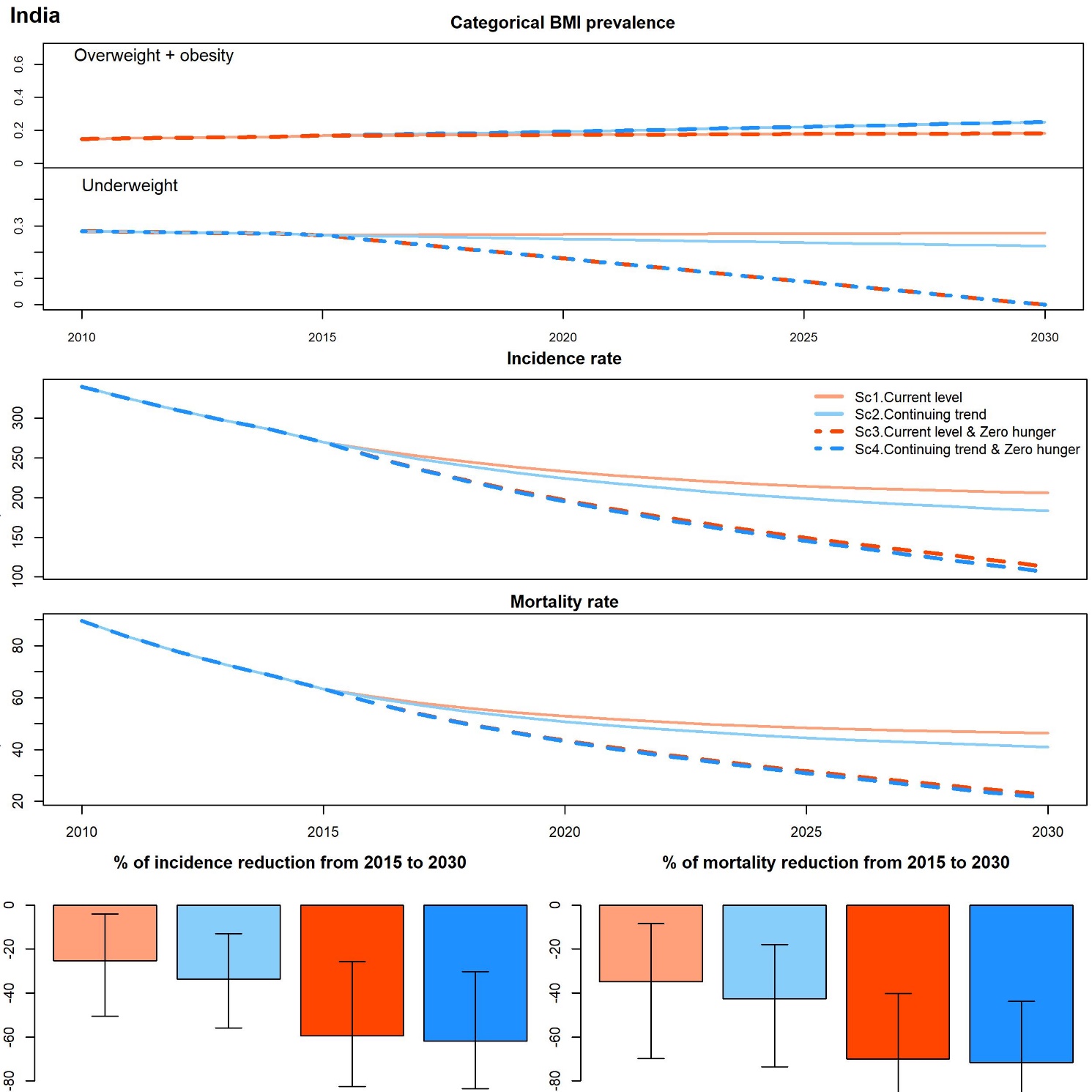
**

**
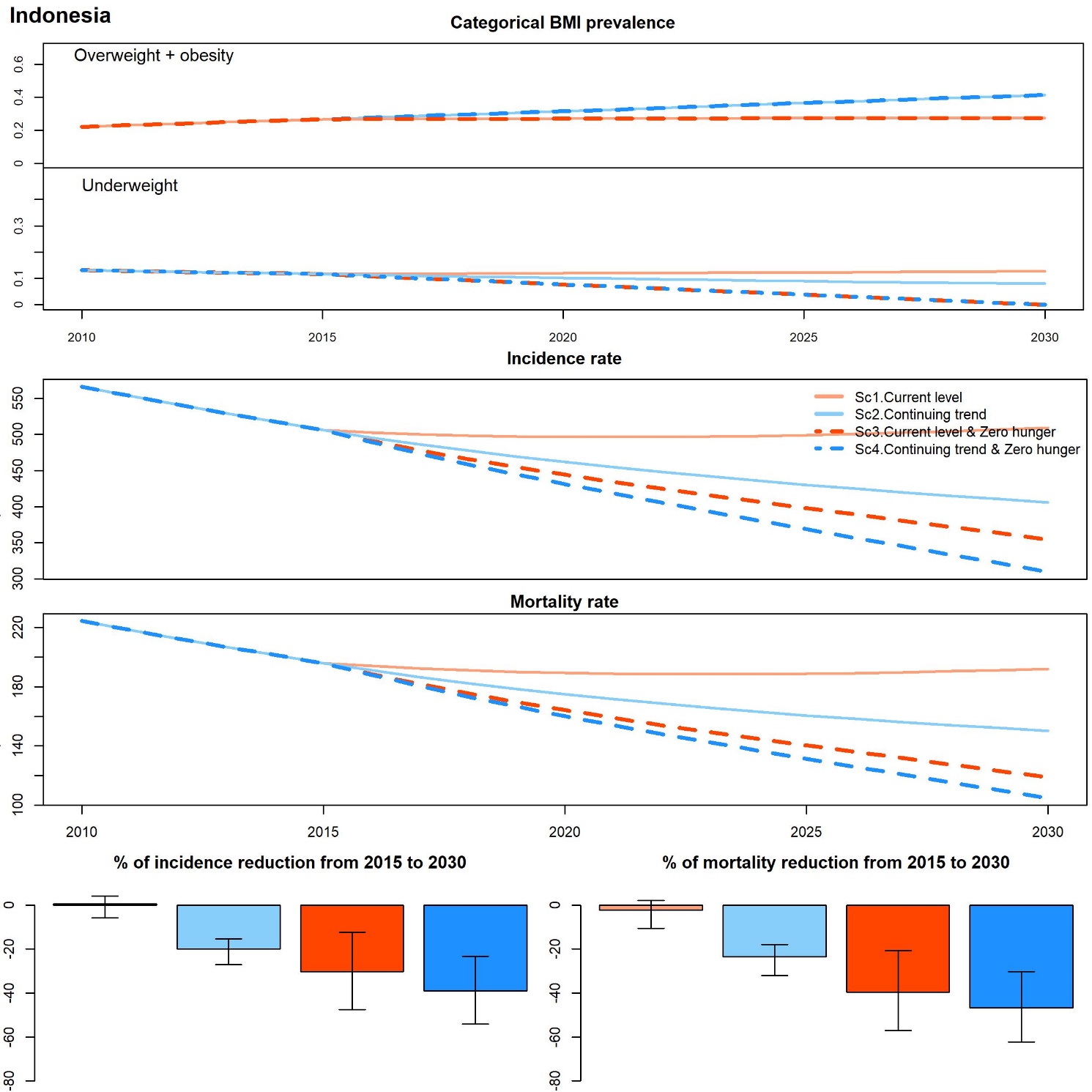
**

**
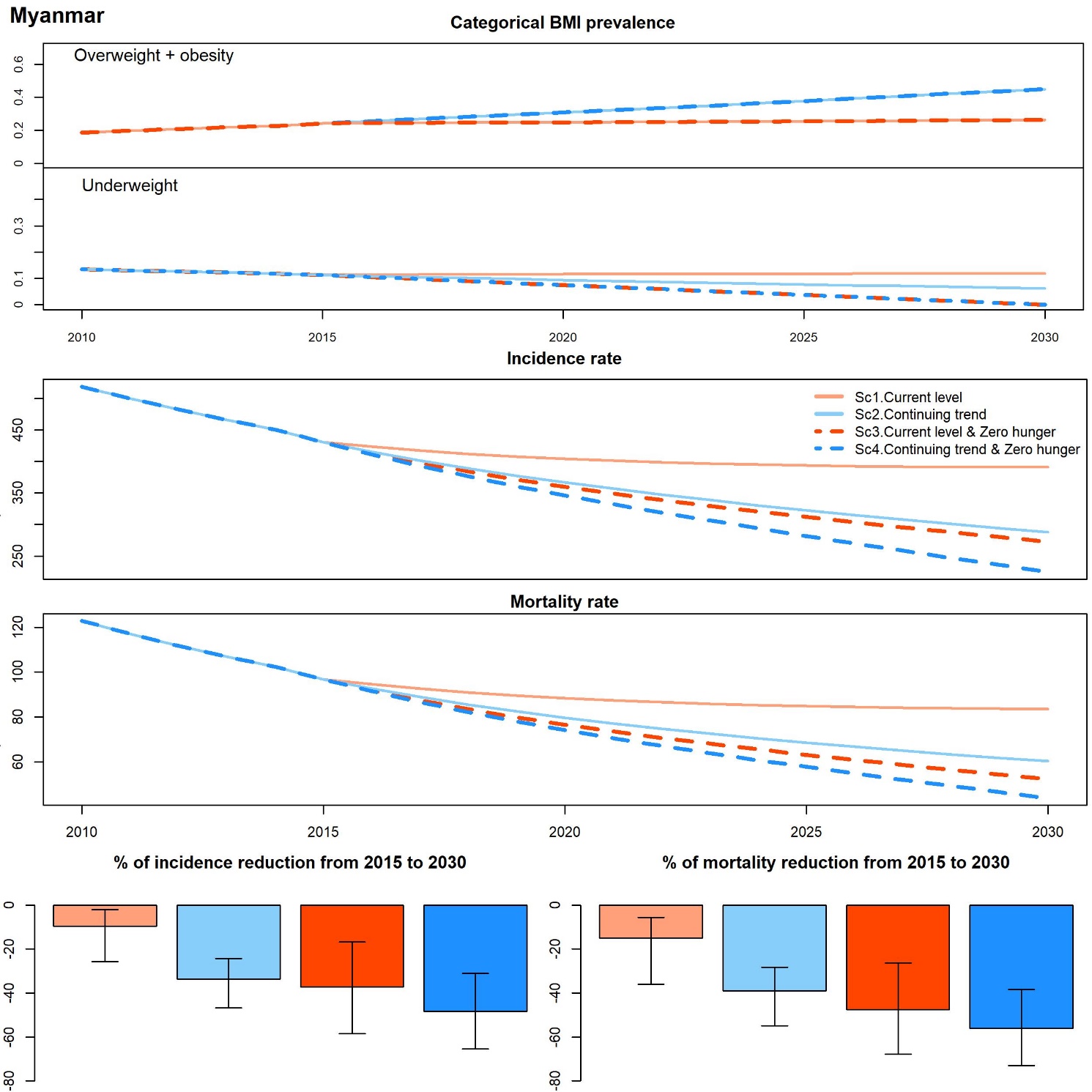
**

**
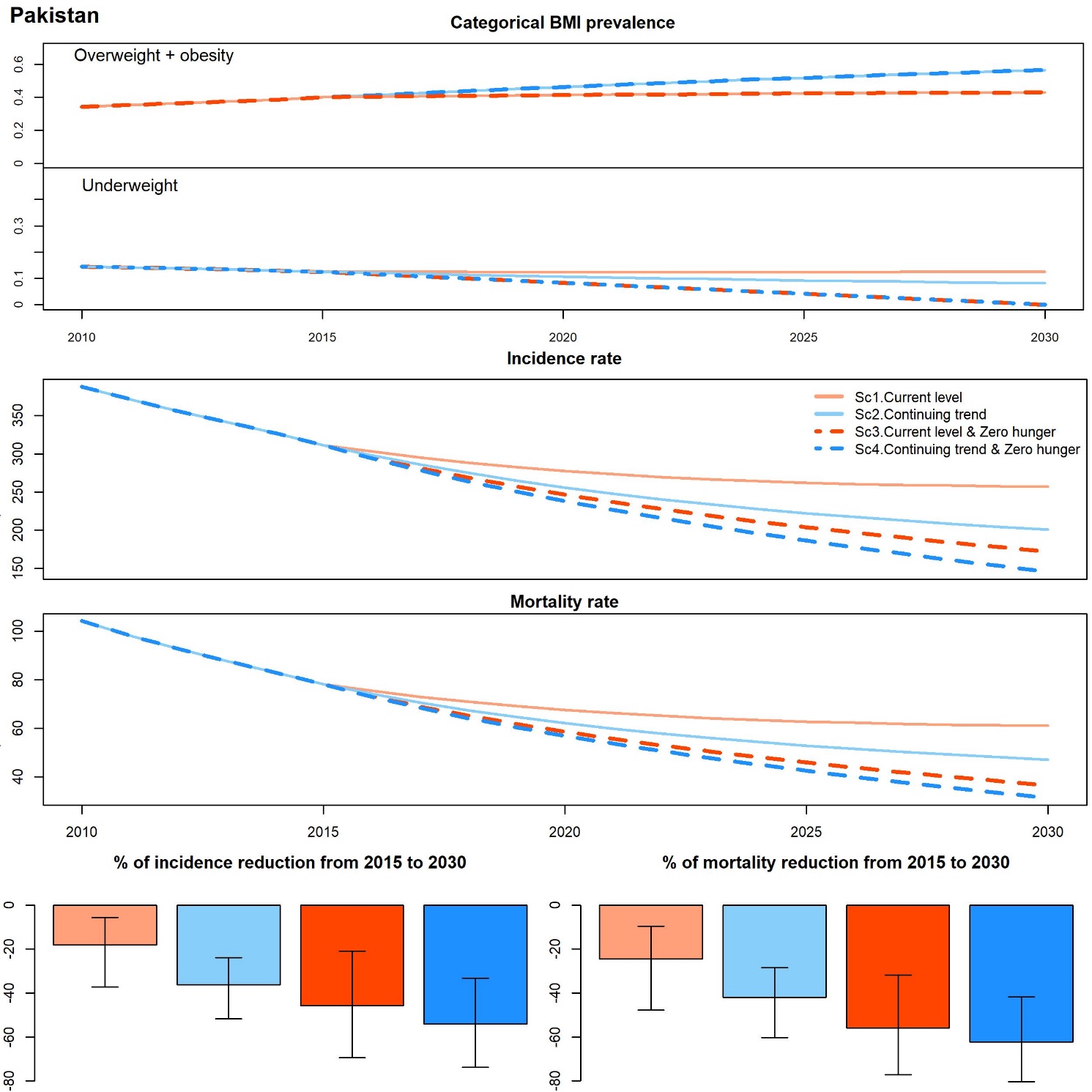
**

**
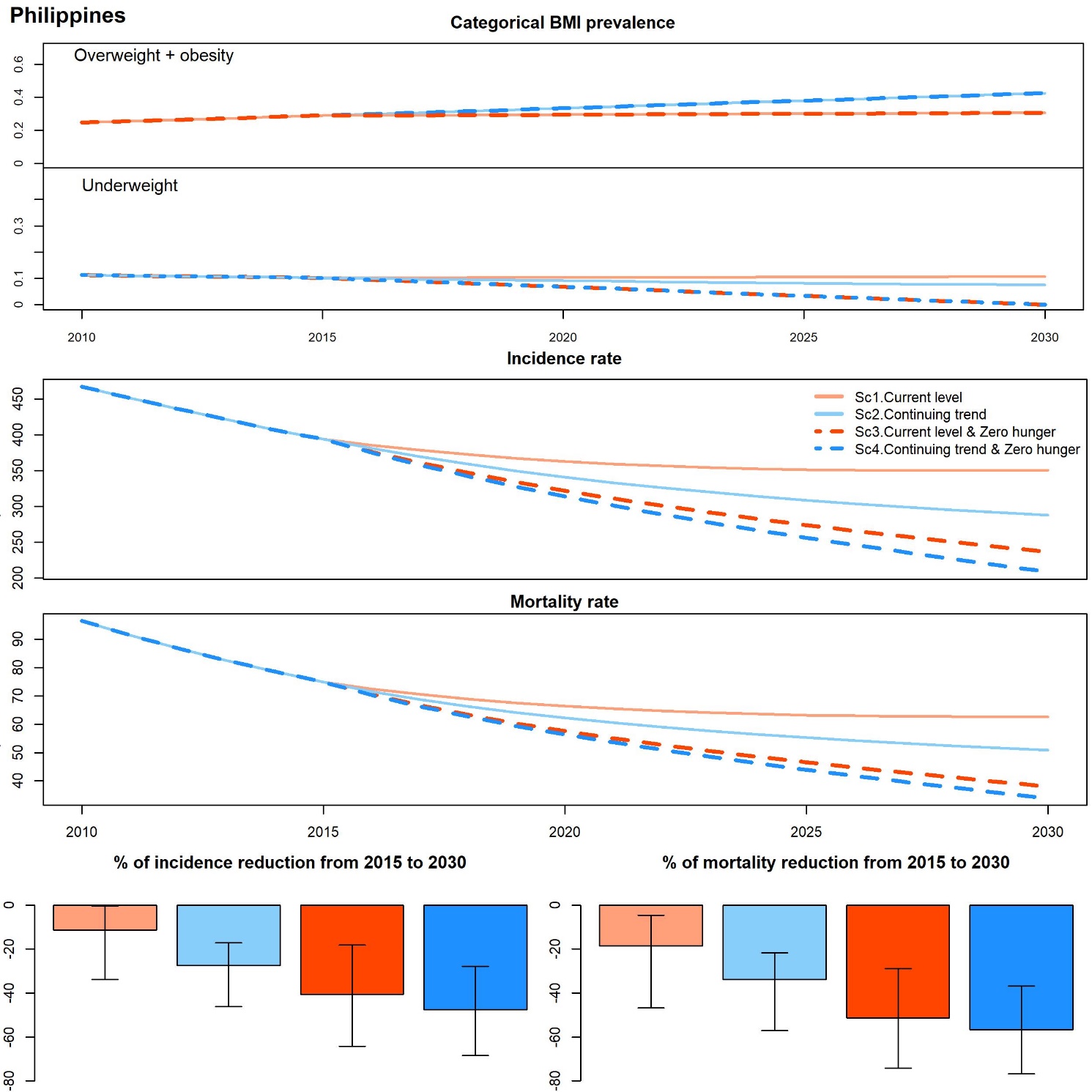
**

**
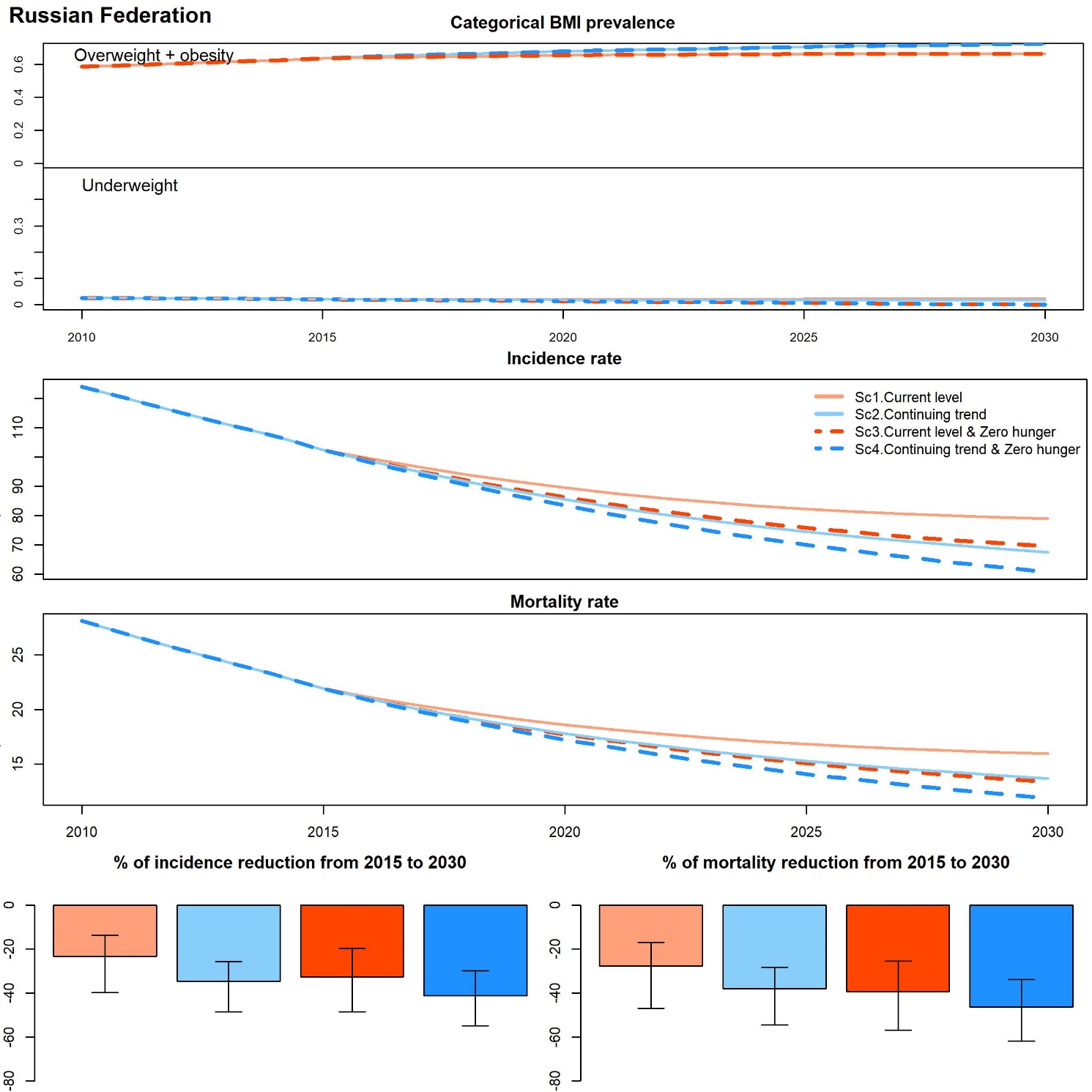
**

**
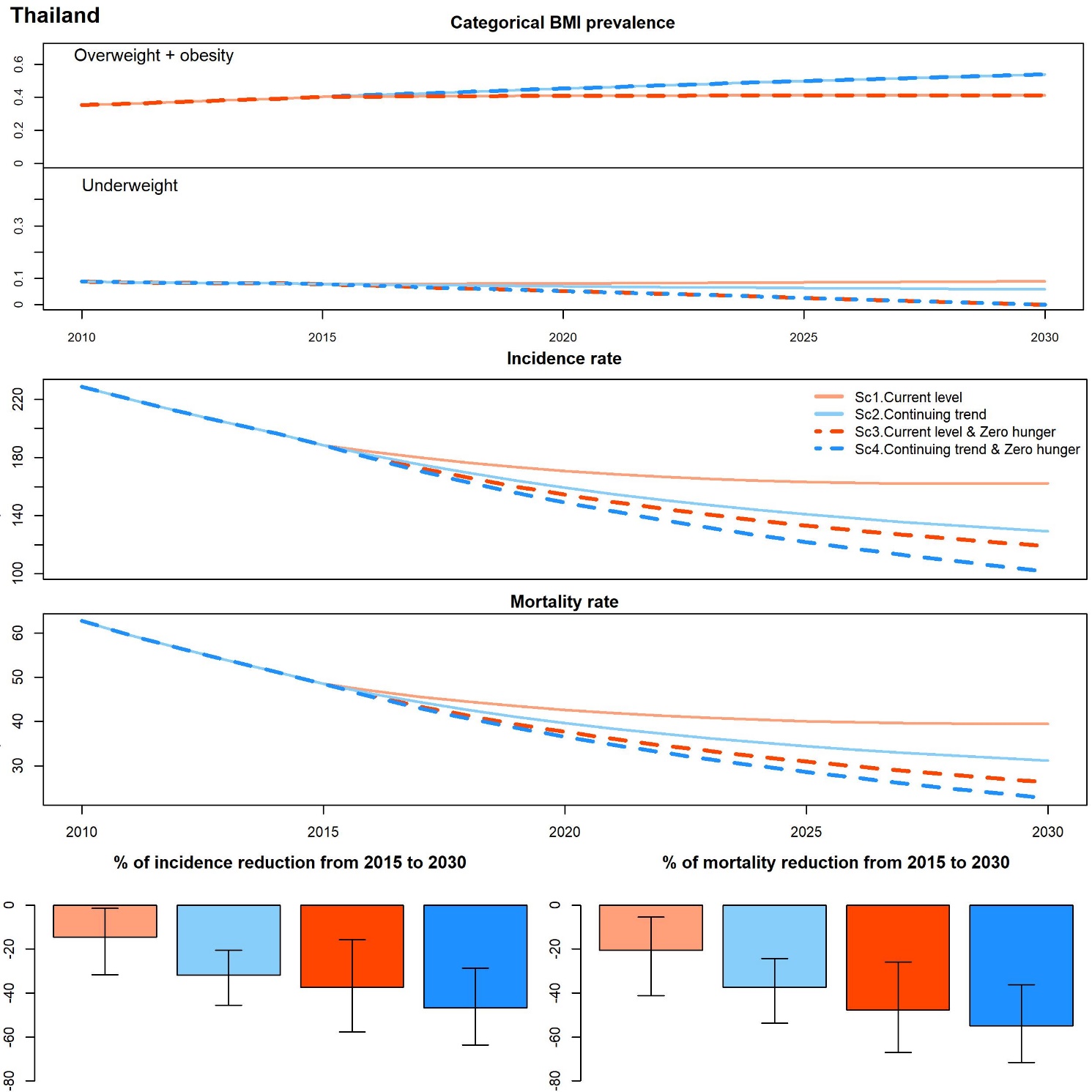
**

**
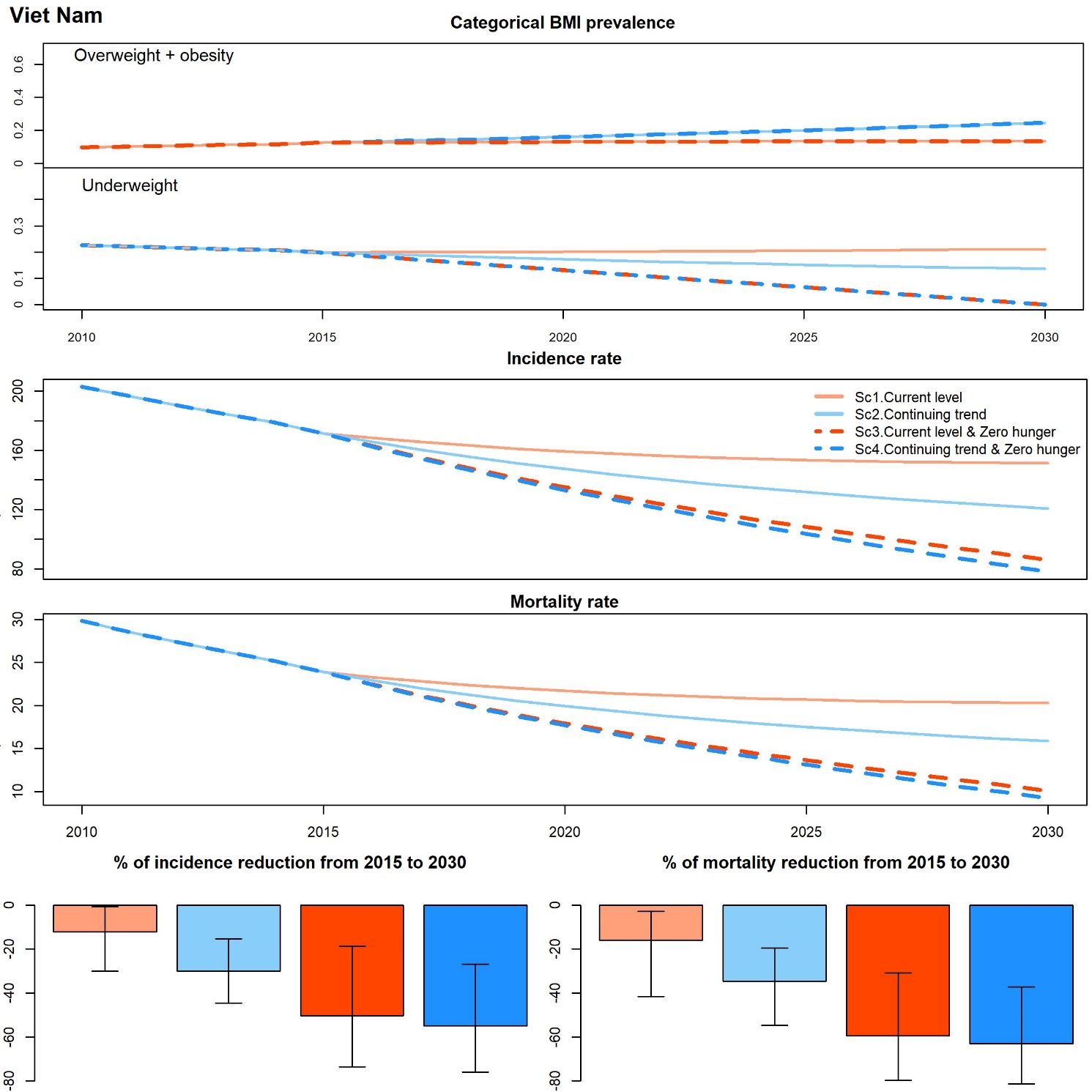
**

**Appendix 5. Sensitivity**

We conducted a one-way sensitivity analysis to examine the impact of each parameter on model outcomes. The analysis was based on posterior parameter distributions, with the minimum value set as the lower bound and the maximum value as the upper bound. We focused on the percentage reduction in TB incidence between 2015 and 2030 under the **Current level & Zero hunger** scenario, compared with the **Current level** scenario, in Bangladesh and the Russian Federation. These two countries were selected to present the highest and lowest underweight prevalence in 2014. The results indicated that TB incidence reduction under the **Current level & Zero hunger** scenario, compared to the **Current level** scenario was most sensitive to the parameter of relative risk of TB incidence among underweight individuals compared to those of normal weight, and the transmission rate parameter.


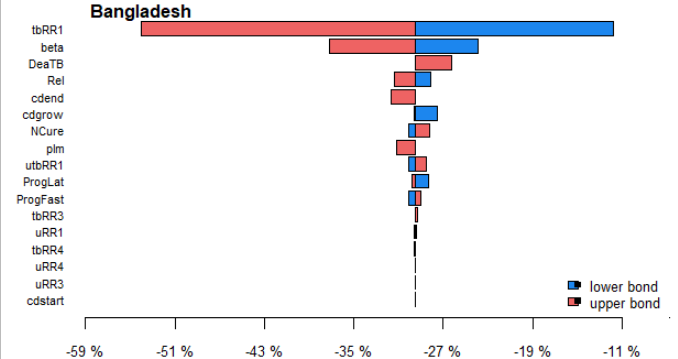


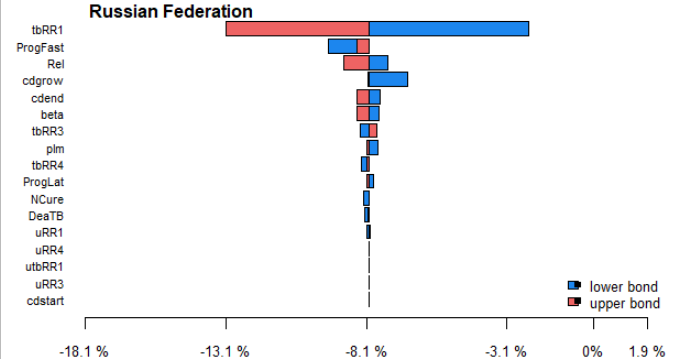


* tbRR1: relative risk of tuberculosis progression rate for underweight

beta: transmission parameter

Rel: relapse rate

utbRR1: relative risk of death rate for active tuberculosis for underweight

DeaTB: death rate for active tuberculosis

Cdgrow: case detection exponential growth rate

ProgFast: progression rate from fast latent

plm: partial immunity

NCure: natural cure rate

tbRR3: relative risk of tuberculosis progression rate for overweight

ProgLat: progression rate from slow latent

Cdend: final level of case detection

tbRR4: relative risk of tuberculosis progression rate for obese

uRR1: relative risk of general mortality for underweight

uRR3: relative risk of general mortality for overweight

uRR4: relative risk of general mortality for obese

cdstart: start level of case detection

**References:**

1. Poole, D. and A.E. Raftery, *Inference for Deterministic Simulation Models: The Bayesian Melding Approach.* Journal of the American Statistical Association, 2000. **95**(452): p. 1244-1255.

2. UNPD, *World Population Prospects: The 2012 Revision.*

3. World Health Organization, *Global Tuberculosis Report 2014.* 2015.

4. NCD Risk Factor Collaboration, *Trends in adult body-mass index in 200 countries from 1975 to 2014: a pooled analysis of 1698 population-based measurement studies with 19· 2 million participants.* The Lancet, 2016. **387**(10026): p. 1377-1396.
