# Supplementary material for: Estimating the impact of nutritional transition and ending hunger on tuberculosis in 12 high-burden countries: a model-based scenario analysis
Source: BMJ Glob Health. 2025 Dec 25;10(12):e018839. doi: 10.1136/bmjgh-2024-018839 (PMC12742055; doi:10.1136/bmjgh-2024-018839)
Supplement: online supplemental file 2 [file bmjgh-10-12-s002.docx]

**Supplemental Figure. Directed acyclic graph (DAG) on the relations between body mass index (BMI), diabetes, and tuberculosis (TB).**

**
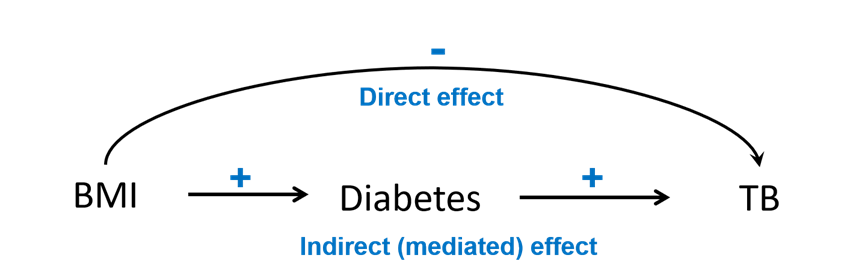
**
