# Supplementary material for: Estimating the impact of nutritional transition and ending hunger on tuberculosis in 12 high-burden countries: a model-based scenario analysis
Source: BMJ Glob Health. 2025 Dec 25;10(12):e018839. doi: 10.1136/bmjgh-2024-018839 (PMC12742055; doi:10.1136/bmjgh-2024-018839)
Supplement: online supplemental file 3 [file bmjgh-10-12-s003.docx]

**Supplemental Table. Model parameter values and prior distributions**

| **Notation** | **Parameter** | **Prior distribution** | **Unit** | **Source** |
| --- | --- | --- | --- | --- |
| $\beta$ | Transmission parameter | uniform(1,30) |  | Fitted to notification data from WHO,2016 |
| $k$ | Rate of leaving fast infection into slow infection | 0.2 | 1/year | Vynnycky et al, 1997 [1] |
| $\mathrm{nc}$ | Natural cure rate | 0.2 (0.1,0.3) | 1/year | Dye et al, 1998 [2] |
| $\mathrm{utb}$ | Death rate for active TB | 0.2 (0.1,0.3) | 1/year | Adapted from Hughes et al. [3] |
| $p$ | Progression rate from fast infection | 0.03 (0.015,0.045) | 1/year | Vynnycky et al, 1997 [1] |
| $\mathrm{ra}$ | Progression rate from slow infection | 0.0003 (0.00015,0.00045) | 1/year | Vynnycky et al, 1997 [1] |
| $e$ | Relapse rate | 0.01 (0.005,0.015) | 1/year | Assumption |
| $\mathrm{im}$ | Partial immunity | 0.79 (0.73,0.85) | - | Andrew et al, 2012 [4] |
| ${tbRR}_{i}$ | Relative risk of TB progression rate by BMI group (i) | i=1, 3.83 (1.53,9.58)  i=2, 1 (ref)  i=3, 0.46 (0.34,0.63)  i=4, 0.30 (0.23,0.37) |  | Dose-response meta-analysis from three cohorts[5, 6]. |
| ${utbRR}_{i}$ | Relative risk of death rate for active TB by BMI group (i) | i=1, 1.71 (1.06,2.35)  i=2, 1  i=3, 1  i=4, 1 |  | Meta-analysis from two studies, Zachariah et al[7] and Yen et al[8]. |
| ${uRR}_{i}$ | Relative risk of general mortality by BMI group (i) | i=1, 1.49 (1.39,1.55)  i=2, 1  i=3, 1.11 (1.10,1.11)  i=4, 1.64 (1.61, 1.67) |  | The Global BMI Mortality Collaboration[9] |

**References:**

1. Vynnycky E, F.P., *The natural history of tuberculosis: the implications of age-dependent risks of disease and the role of reinfection.* Epidemiology and infection, 1997. **119(02)**: p. 183-201.

2. Dye C, G.G., Sleeman K, Williams BG, *Prospects for worldwide tuberculosis control under the WHO DOTS strategy. Directly observed short-course therapy.* Lancet 1998. **52(9144)**: p. 1886-1891.

3. Hughes G, C.C., Corbett EL, *Modeling tuberculosis in areas of high HIV prevalence.* Proceedings of the 38th conference on Winter simulation, 2006: p. 459-465.

4. Andrews JR, N.F., Walensky RP, Cerda R, Losina E, Horsburgh CR, *Risk of Progression to Active Tuberculosis Following Reinfection With Mycobacterium tuberculosis.* Clinical Infectious Diseases, 2012. **54(6)**: p. 784-91.

5. Cegielski, J.P., L. Arab, and J. Cornoni-Huntley, *Nutritional risk factors for tuberculosis among adults in the United States, 1971-1992.* Am J Epidemiol, 2012. **176**(5): p. 409-22.

6. Lin, H.H., et al., *Association of obesity, diabetes, and risk of tuberculosis: two population-based cohorts.* Clin Infect Dis, 2017.

7. Zachariah, R., et al., *Moderate to severe malnutrition in patients with tuberculosis is a risk factor associated with early death.* Trans R Soc Trop Med Hyg, 2002. **96**(3): p. 291-4.

8. Yen, Y.-F., et al., *Association of Body Mass Index With Tuberculosis Mortality: A Population-Based Follow-Up Study.* Medicine, 2016. **95**(1): p. e2300.

9. Global, B.M.I.M.C., et al., *Body-mass index and all-cause mortality: individual-participant-data meta-analysis of 239 prospective studies in four continents.* Lancet, 2016. **388**(10046): p. 776-86.
